# A high-resolution pediatric female whole-body numerical model with comparison to a male model

**DOI:** 10.1088/1361-6560/aca950

**Published:** 2023-01-13

**Authors:** Georgios Ntolkeras, Hongbae Jeong, Lilla Zöllei, Adam A Dmytriw, Ali Purvaziri, Michael H Lev, P Ellen Grant, Giorgio Bonmassar

**Affiliations:** 1 Fetal-Neonatal Neuroimaging and Developmental Science Center, Division of Newborn Medicine, Boston Children’s Hospital, Boston, United States of America; 2 Department of Pediatrics, Baystate Medical Center, Springfield, United States of America; 3 Athinoula A. Martinos Center for Biomedical Imaging, Massachusetts General Hospital, Charlestown, United States of America; 4 Department of Radiology, Boston Children’s Hospital, Boston, United States of America; 5 Department of Radiology, Massachusetts General Hospital, Boston, United States of America

**Keywords:** EM simulation, 7 Tesla MRI, SAR, medical device, MRI safety, gender, Sim4Life

## Abstract

**Objective.:**

Numerical models are central in designing and testing novel medical devices and in studying how different anatomical changes may affect physiology. Despite the numerous adult models available, there are only a few whole-body pediatric numerical models with significant limitations. In addition, there is a limited representation of both male and female biological sexes in the available pediatric models despite the fact that sex significantly affects body development, especially in a highly dynamic population. As a result, we developed Athena, a realistic female whole-body pediatric numerical model with high-resolution and anatomical detail.

**Approach.:**

We segmented different body tissues through Magnetic Resonance Imaging (MRI) and Computed Tomography (CT) images of a healthy 3.5 year-old female child using 3D Slicer. We validated the high anatomical accuracy segmentation through two experienced sub-specialty-certified neuro-radiologists and the inter and intra-operator variability of the segmentation results comparing sex differences in organ metrics with physiologic values. Finally, we compared Athena with Martin, a similar male model, showing differences in anatomy, organ metrics, and MRI dosimetric exposure.

**Main results.:**

We segmented 267 tissue compartments, which included 50 brain tissue labels. The tissue metrics of Athena displayed no deviation from the literature value of healthy children. We show the variability of brain metrics in the male and female models. Finally, we offer an example of computing Specific Absorption Rate and Joule heating in a toddler/preschooler at 7 T MRI.

**Significance.:**

This study introduces a female realistic high-resolution numerical model using MRI and CT scans of a 3.5 year-old female child, the use of which includes but is not limited to radiofrequency safety studies for medical devices (e.g. an implantable medical device safety in MRI), neurostimulation studies, and radiation dosimetry studies. This model will be open source and available on the Athinoula A. Martinos Center for Biomedical Imaging website.

## 1. Introduction

Computational modeling with virtual humans is used to study the interaction of complex biological problems in silico and minimize the *in vivo* experimental studies, which is especially important in children (Council 2006). The spectrum of applications of numerical models includes analyses of electric or magnetic source localization, dosimetry, radiofrequency (RF) and specific absorption rate (SAR) exposure, neurostimulation, anatomic implant development, as well as industries involving accident simulations (e.g. crashes or blasts) and clothing (Gosselin *et al* 2014, Jeong *et al* 2021a). As a result, the accurate anatomical representation of human numerical models has become integral to many state-of-the-art safety studies and medical device developments. The complexity of a numerical model should be able to represent the degree of detail needed from the experimental process where it is used, providing a realistic representation of the *in vivo* experimental process and allowing the operators to adjust the experimental parameters (Gosselin *et al* 2014).

The physiological and anatomical systems of the human body are inherently complex, and their translation to numerical models has significantly evolved in the past years. In fact, since 1950, when the first generations of computational models were introduced, there have been advances in model design and fabrication to meet the increasing needs in the medical and other industries (Gosselin *et al* 2014). Although there is still a significant lack of high-resolution and anatomically accurate numerical models for the toddler/preschool age, the existing models have significant limitations: (a) morphing, (b) low level of detail, (c) and poor validation techniques (Jeong *et al* 2021a). A closer look at the literature indicates that the two models of interest of the *Virtual family (or Virtual Population)* have significant limitations, including but not limited to the lack of detailed information about the brain. In particular, Nina, a 3 year-old female model, has a limited number of segmented tissues (97 body tissues) and was developed by morphing Roberta, a 5 year-old, leading to anatomical inaccuracies given the non-proportional growth of the organs of the body during childhood (Gosselin *et al* 2014). In addition, a model now part of the GSF family known as *the child*, a 7 year-old girl, was segmented based only on CT images which significantly limits its level of detail in the soft tissues (e.g. brain instead of white matter, gray matter) while the cortical bone was not differentiated from the bone marrow as well (Petoussi-Henss *et al* 2002).

Furthermore, a different model developed by Lee *et al* (2009), the Korean child model, is an older (7 year-old) child whose segmentation underwent significant morphing, was based only on Magnetic Resonance Imaging (MRI), and had limited spatial resolution (i.e. 1 mm × 1 mm × 3 mm) (Lee *et al* 2009). The UF family was the first group of body models comprised of 4 and 8 year-old children who were only head-to-torso models. The segmentation was only based on Computed Tomography (CT) images (Lee *et al* 2005). Later, in 2010, added models of 1, 5 and 10 year-old children were segmented based only on CT images that also did not include the arms, while the cervical spine was based on CT datasets of a 15 year-old girl, which introduced significant resizing and morphing to the final result (Lee *et al* 2010). The arms were later added based on images obtained from an 18 year-old cadaver. The series of 92 pediatric extended cardiac-torso (XCAT) models included ages ranging from newborn to 15 year-old that were segmented based on Positron Emission Tomography—CT datasets that did not include the arms and legs. The extremities were taken from previously developed models, but they were resized, morphed, and manually attached in order to fit the developed models. Furthermore, some CT scans had incomplete skulls (Norris *et al* 2014, Segars *et al* 2015). Finally, the *Chinese family* models of 5 and 10 year-old males were based only on previously developed *adult* models that were morphed (Zhang *et al* 2009, Pi *et al* 2018).

The lack of toddler/early preschooler-age pediatric models with high tissue resolution and accurate anatomy led our team to build Martin, a 29 month-old male numerical model based on MRI and CT (Jeong *et al* 2021a). Although, to the best of our knowledge, the female population of the 1–4 years age range is still significantly underrepresented. Given the anatomy of the various developmental stages, it is crucial to numerically model both males and females equally due to the prominent biological and anatomical differences between the sexes. Those differences are highlighted by the Centers for Disease Control (CDC) and Prevention, which uses different charts for the growth trajectory of boys and girls, including but not limited to metrics such as weight, height, and head circumference (Cdc 2000, National Center for Chronic Disease Prevention and Health Promotion 2000). Further literature indicates that individual body organs have different sizes and weights in children of the same age but opposite gender. Also, the genital organs are unique for each gender, and only separate models for males and females can reflect those differences (Chang *et al* 2021). The National Institute of Health (NIH) now has active policies that ensure the inclusion of women in NIH-funded research to account for sex as a Biological Variable (NIH Policy on sex as a biological variable, NIH 2020: https://orwh.od.nih.gov/sex-gender/nih-policy-sex-biological-variable#:~:text=NIH, Inclusion of Women and Minorities as Participants in Research Involving Human Subjects. Available from: https://grants.nih.gov/policy/inclusion/women-and-minorities.htm, Arnegard *et al* 2020). As a result, it is highly significant for both sexes to be represented in numerical models to allow for studies of sex as a biological variable in young children.

This manuscript presents Athena, a state-of-the-art 3.5 year-old female, whole-body, high-resolution, and anatomically accurate numerical model segmented directly from MRI and CT images of a healthy subject, which was selected based on the 50th percentile of the CDC charts for weight and height. The Athena model was based on a high-quality and larger imaging dataset, including full-body MRI T1 and inversion recovery (IR) images and CT imaging data that allowed for more detailed segmentation of specific body tissues (e.g. blood vessels). The model validation was performed by comparing literature values, studying the output of different segmentors, and by continuous feedback from expert pediatric radiologists.

Finally, although the United States (US) Food and Drug Administration cleared the first seven-Tesla (7 T) MRI device in 2017, the use of 7 T MRI systems for patients who weigh less than 66 pounds has still not been cleared (Caccomo 2017). Only a few MRI safety studies have been conducted up to this point at 7 T (Malik *et al* 2021). This study illustrates how to employ the Athena and Martin models for MRI safety studies by comparing results on EM ${\text{B}}_{1}^{+}$.transmit magnetic field exposure and the thermal estimations at 7 T for the two sexes. With the increased interest in computational modeling, these models are expected to be used in various studies, including MRI safety. Athena will be an open-source, freely distributed model available on the Athinoula A. Martinos Center for Biomedical imaging website.

The main contributions of this paper are:

1. The development and free distribution of Athena, a state-of-the-art 3.5 year-old female, whole-body, 0.5 × 0.5 × 0.5 mm resolution, and anatomically accurate numerical model with 267 tissue compartments.
2. Examples include anatomical, organ metrics, and MRI dosimetry to account for sex as a biological variable.

## 2. Material and methods

### 2.1. Subject and data acquisition

**Figure 1.**
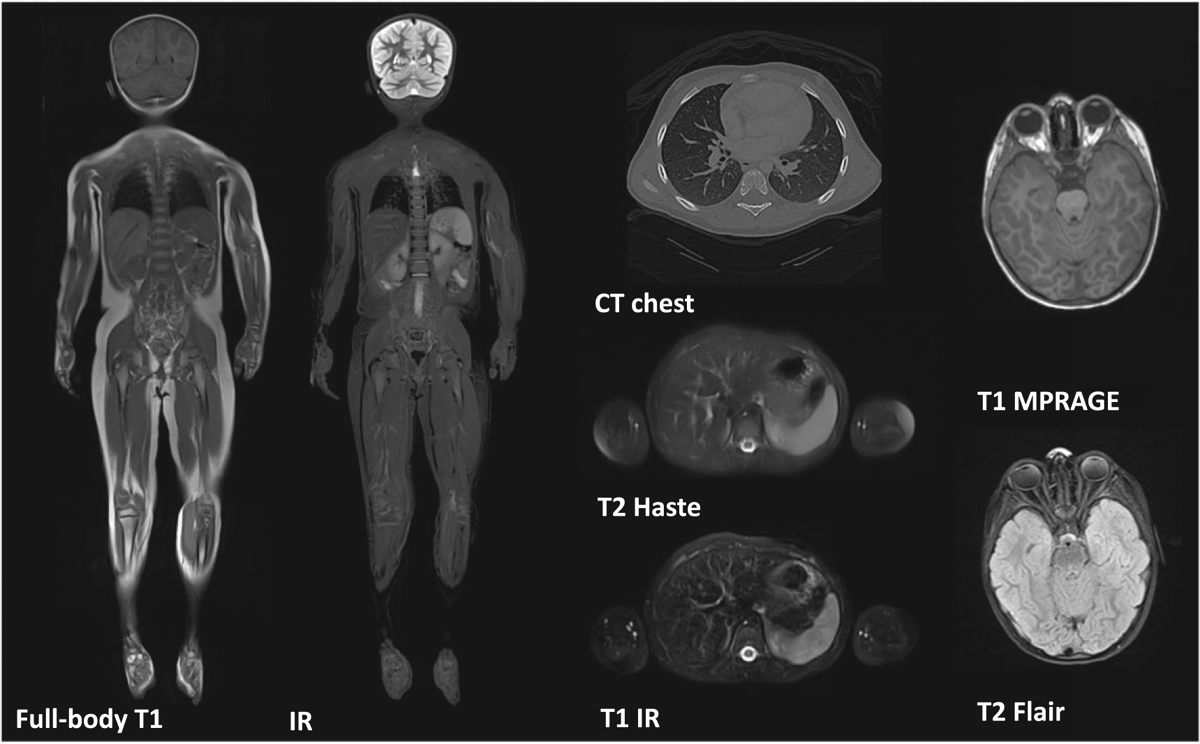
Structural MRI and CT scans used for the segmentation process. Different scans are used to visualize the body tissues with various levels of detail. We used full body T1 and IR images to segment the female model’s tissues from head to toes. In addition, we used T2 HASTE, T1 IR, and CT images of the chest and upper abdomen to extract further information about the anatomical structures of this body compartment. We also used T1 MPRAGE and T2 Flair Images for the brain segmentation.

**Figure 2.**
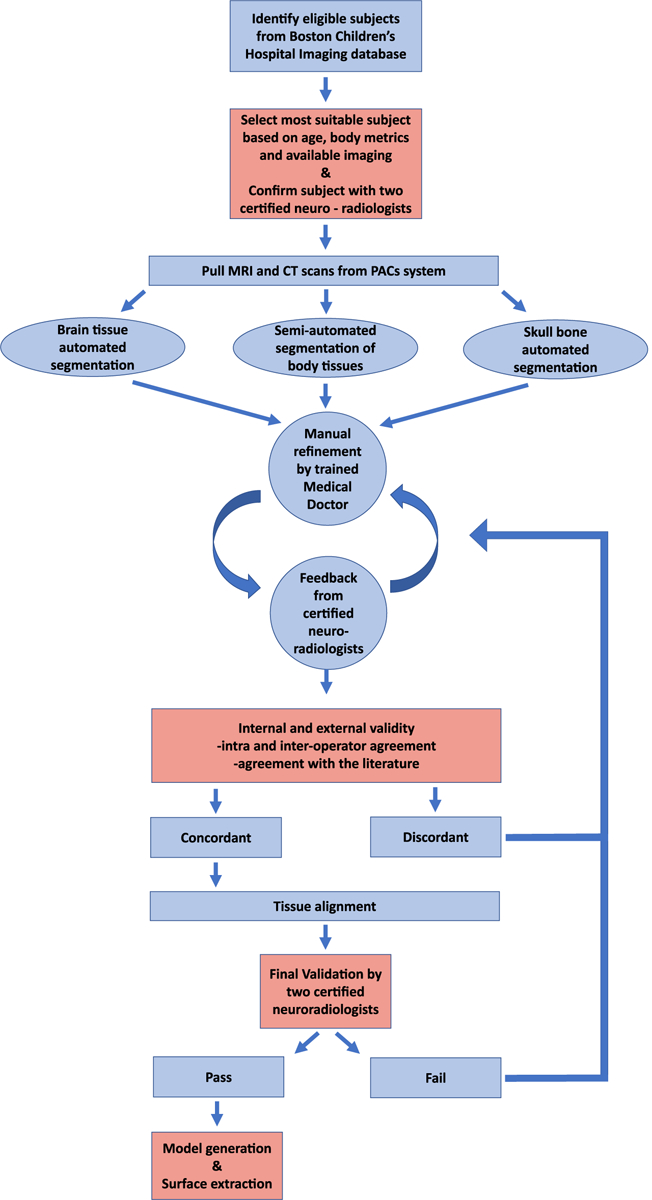
Flow Diagram—model development: we used Boston Children’s Hospital patient database to identify eligible subjects, and we selected the subject that better fit the purposes of this study after evaluating all the eligible subjects with the two specialized neuroradiologists. We pulled all available MRI and CT scans performed at the age of interest and initiated the segmentation process using different tools depending on the tissue of interest. We manually refined the initial result through a feedback-loop process with the input of the senior neuroradiologists. The outcome was then validated using objective measurements, and the tissues that passed this process were aligned in the same 3D space. If a tissue failed to pass any step, manual refinement was applied, and the validation process was repeated. The result underwent a final validation before extracting the surfaces of each tissue and generating the model.

A 3.5 year-old female child was selected based on the availability of multiple imaging sequences, image quality, and the lack of anatomical abnormalities (figure 1) that would facilitate the segmentation process (figure 2). Two certified neuroradiologists with more than 20 years of experience, P E G and M H L, assessed the image quality and the lack of anatomical abnormalities. The clinical report from each imaging study confirmed the absence of any anatomical abnormality. We selected the subject that we used to develop the female toddler numerical model based on its body metrics after establishing that it was a representative subject of the female children of her age (height: 95.4 cm, 50th percentile, and weight:14.7 kg, 52nd percentile at the time of the MRI) (Cdc 2000). Images were retrieved from Boston Children’s Hospital’s Picture Archiving and Communication System (PACS) database. The study protocol received approval from the Boston Children’s Hospital (BCH) Institutional Review Board, which waived the need for written informed consent due to the study’s secondary use of data in compliance with the Health Insurance Portability and Accountability Act (HIPAA).

### 2.2. Data processing and registration

**Figure 3.**
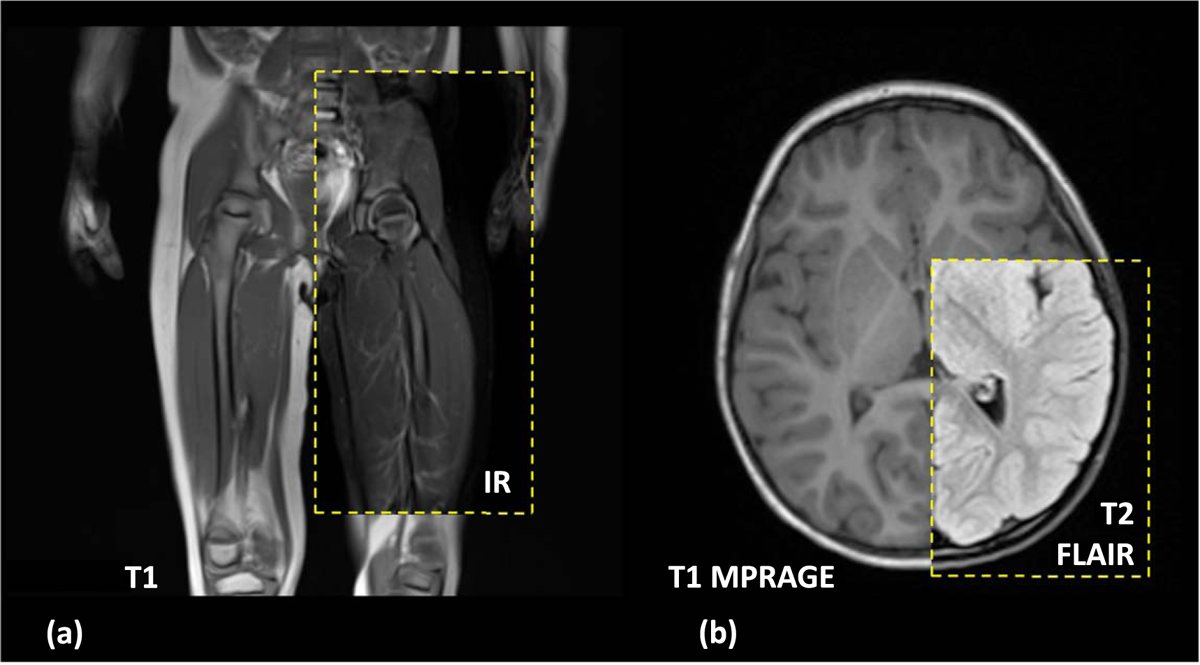
Registration of different MRI scans: (a) coronal view of T1 MRI registered with IR sequence, the contrast of vessels and intervertebral discs was enhanced in IR scans. (b) Axial view of T1 MRRAGE sequence registered with T2 Flair sequence where fat suppression is shown in the subcutaneous fat and the myelinated white matter tracts.

Tissue segmentation labels were reviewed on different MRI sequences (T1, IR, MPRAGE, T2 Haste, T2 FLAIR) that covered all the body from head to toes, including all four extremities, as well as CT data from the chest and lower neck of the same subject (figure 1, table 1). Medical images were resampled to 0.5 × 0.5 × 0.5 mm^3^ using a Lanczos interpolation method (Duchon 1982, Bentbib *et al* 2016, Jeong *et al* 2021a). Co-registration between different sequences and modalities was performed using an extension tool in 3D Slicer (figure 3). For the MRI sequences, linear registration (six affine degrees of freedom as rotation in *x*, *y*, and *z* and translation in *x*, *y* and *z*) was used to align images using the whole-body coronal T1 image as a reference volume. Nonlinear registration was done to align the CT image into the reference MRI image volume using Elastix’s 3D Slicer extension tool (Fedorov *et al* 2012). For the detailed process, please see the process described in the previous work of the Martin model (Jeong *et al* 2021a).

### 2.3. The segmentation

**Figure 4.**
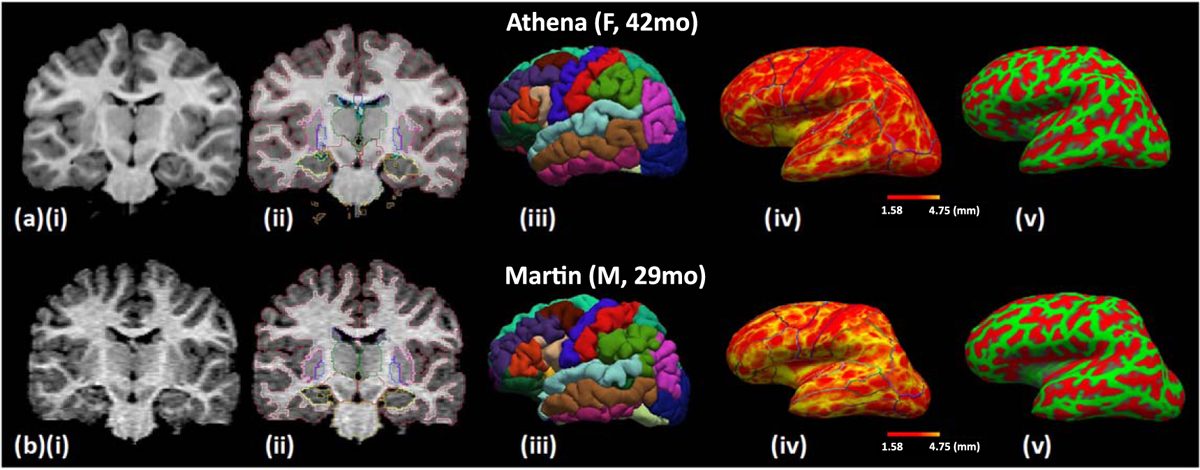
Brain automatic segmentation. (a) Brain segmentation of Athena a 42 months old (mo) female child (i) T1 brain MRI scan used for segmentation purposes. (ii) Automated tool outlining the brain anatomical structures based on established anatomical atlas (Desikan-Killiany). (iii) 3D reconstruction of the brain with each color indicating a different anatomical region following the same anatomical atlas. (iv) Gray matter thickness maps with the red area corresponding to lower and the yellow to higher thickness (mm). (v) Curvature map as an index of brain folding with green color corresponding to gyri and red color to brain sulci. (b) The same process was followed the male model (Martin 29 mo) for comparison purposes.

**Figure 5.**
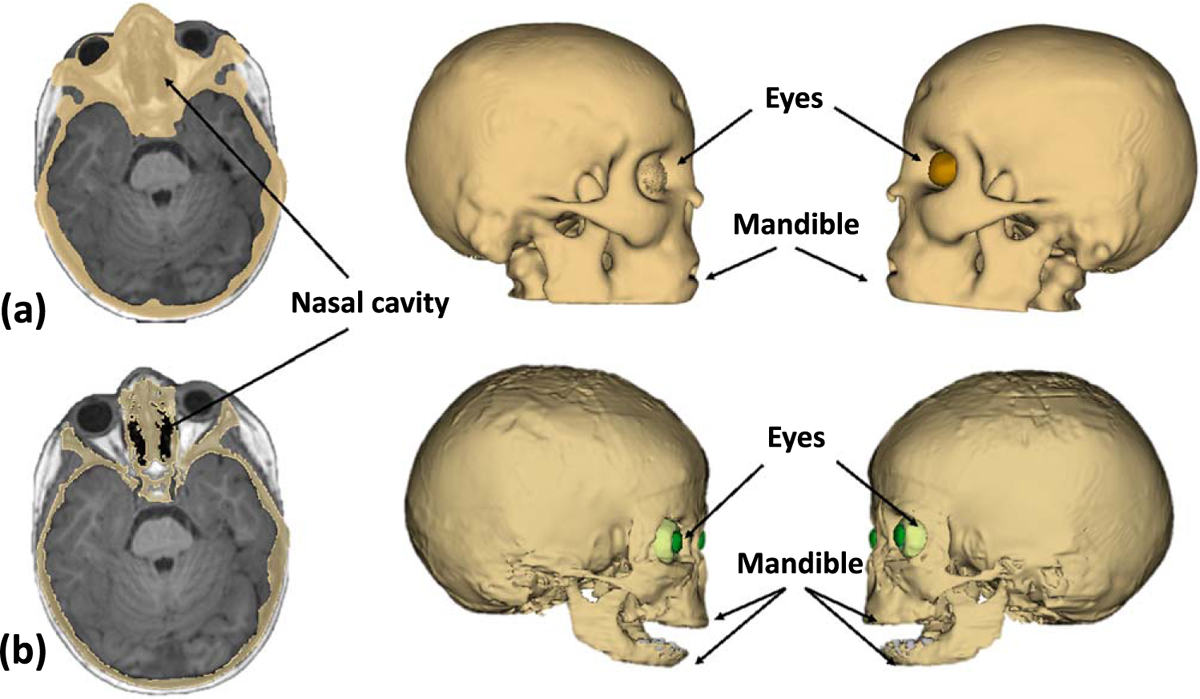
Skull segmentation: (a) skull segmentation using SimNIBS, an automatic segmentation, overestimation of the skull can be seen in areas such as the nasal cavity, the eyes, and the upper and lower mandible. (b) Skull label after manual refinement with the nasal cavity being refined in spaces that include air and mucus while the orbital area was also refined, allowing for the segmentation of additional tissues such as the infraorbital fat, the optic nerves, and the ophthalmic muscles.

A whole-body 3 T MRI scan (Siemens Healthineers, Germany) was used to segment the body tissues, and CT images (Siemens Healthineers, Germany) of the neck and chest were used in combination with the MR images to segment the bones of the torso, while MR images were used to segment the bones of the rest of the body. We used the image computing platform *3D Slicer* (Fedorov *et al* 2012) to segment and visualize the brain and non-brain anatomical regions (Fedorov *et al* 2012). 3D Slicer provides several automated and manual segmentation tools that can perform a high-detail segmentation process of simple and more complex body structures. An automated infant-specific segmentation tool was employed (Zöllei *et al* 2020) to segment the brain structures (e.g. cortex white matter, deep brain structures, and cerebellum), as this tool allows for a better grey-white matter differentiation in the pediatric compared to the adult brains (figures 4(ai), (aii) and, (aiii)). Automated brain segmentation metrics such as gray matter volume, thickness, folding index, brain surface, and curvature were extracted while the same process was applied for an already developed male model Martin for comparison purposes (figures 4(aiv), (av) and (b)). For the skull segmentation, an automated method of segmentation using SimNIBS was followed by manual refinement, for which we used the MRI scans available for the head (T1 MPRAGE, IR, T2 Flair) (figure 5) (Thielscher *et al* 2015). A physician member of our team with experience in pediatric whole-body numerical model development (GN) performed manual segmentation and refinement of the automatically segmented tissues (Jeong *et al* 2021a).

Manual segmentation on 3D slicer software resulted in partial tissue overlap for some of the segmented tissues. In order to address this issue, the tissue segmentation was organized in hierarchical compartments or tissue which had a higher label number for small compartments (e.g. accumbens, caudate). Higher priority was given to the segmentation label with the higher number, thus ignoring overlaps with lower label compartments. The segmentation results were exported and processed again using the iSEG (ZMT, Switzerland) (The Medical Image Segmentation Tool Set iSEG 2018). Any voxel non-assigned to tissue was automatically assigned to an adjacent tissue using a supplant tissue tool, and the skin layer was added with 1.0 mm thickness to the segmentation volume as indicated by ICRP Publ. 89 (ICRP 2002).

The surface generation tool Sim4Life (ZMT, Switzerland) was used to create surface meshes for data size reduction without self-interactions and manifold.

### 2.4. The validation

All segmented tissues were reviewed by the two subspecialized neuroradiologists (P E G and M H L) in different stages of the model development process to ensure that the segmentation had the maximum anatomical accuracy and was representative of the female population at this age. Tissues were refined based on the experts’ feedback, and further quality control was performed, assessing the intra- and inter-operator variability and the consistency with literature-reported measurements for specific tissues. For the calculation of the inter- and intra- operator variability the gold standard was generated by feedback and refinement of the segmentation result of the primary segmentor (GN) by the two neuroradiologists P E G and M H L. Finally, all the segmented tissues were visualized together in the same 3D space of the full-body T1 MRI image. Overlapping and misalignment correction was manually performed, and the two neuroradiologists did the final validation. This workflow process was similar to the process followed by our team for developing the male pediatric model since it provides a multilayer validation approach and leads to an optimal, anatomically accurate, and realistic segmentation result. For the heart, given the challenge of its shape variation with time, all three segmentors (GN, AD, and AP) came to an agreement on basic anatomical landmarks (e.g. the apex and the large vessels’ insertion site) before they performed their individual segmentation.

The inter-operator variability was evaluated using the Dice similarity coefficient (DSC) index and the Hausdorff distance (H-d). The DSC was defined as DSC = 2|*X* ∩ *Y*|/(|*X*|+|*Y*|) where is the manually segmented region by one of the segmentors and *Y* is the gold standard (GS) tissues segmentation result. The gold standard was generated by feedback and refinement of the segmentation result of the primary segmentor (GN) by the two neuroradiologists P E G and M H L. The H-d was introduced to overcome the limitation of the DSC index in evaluating tissues with large volumes (e.g. skin, long bones, lungs, liver, kidneys), measuring the distance between segmentation results of the three segmentors and the gold standard in a total of seventeen different subsets of tissues. The two subspecialized neuroradiologists edited, reviewed, and approved the gold standard. The dimensions of selected representative segmented tissues were compared with literature values (Robinow and Chumlea 1982, Chang *et al* 2021). To calculate the weight of each organ, we multiplied the tissue density taken for the Information Technologies In Society (IT’IS) foundation by its tissue volume segmented with 3D slicer (Hasgall *et al* 2022). A ‘pass or fail’ method was used to validate each segmented tissue, which was finalized when both subspecialized neuroradiologists scored the tissue with a ‘pass’ (figure 2).

### 2.5. Statistics

Descriptive statistics were used to describe the metrics acquired from the automatic segmentation of the brain of Athena and Martin numerical models, and the results are reported as mean values with standard deviation (SD), (mean, ±SD).

### 2.6. Example of MRI radiofrequency (RF) safety simulation

Age-dependent tissue properties (e.g. electrical conductivity, relative permittivity, and tissue perfusion rate) were adjusted to the 3.5 year old’s tissue properties by applying the age-dependent conversion ratio to the adult properties (Hasgall *et al* 2022). The age-dependent conversion ratio for the electrical conductivity and permittivity was estimated based on Peyman’s study (also see formula S-1) (Peyman *et al* 2002). Furthermore, the perfusion rate was adjusted using the value reported by Chang *et al* (2021). Previous studies used an averaged age-dependent conversion ratio for the tissues without age dependency measurement data. The tissue properties of liquid tissues (e.g. blood, cerebrospinal fluid (CSF), eye vitreous humor, intestine/stomach contents, Urine) were considered not to change across ages (Dimbylow *et al* 2010). Tissue density, thermal conductivity, and specific heat capacity were considered not to have any age-dependency.

**Figure 6.**
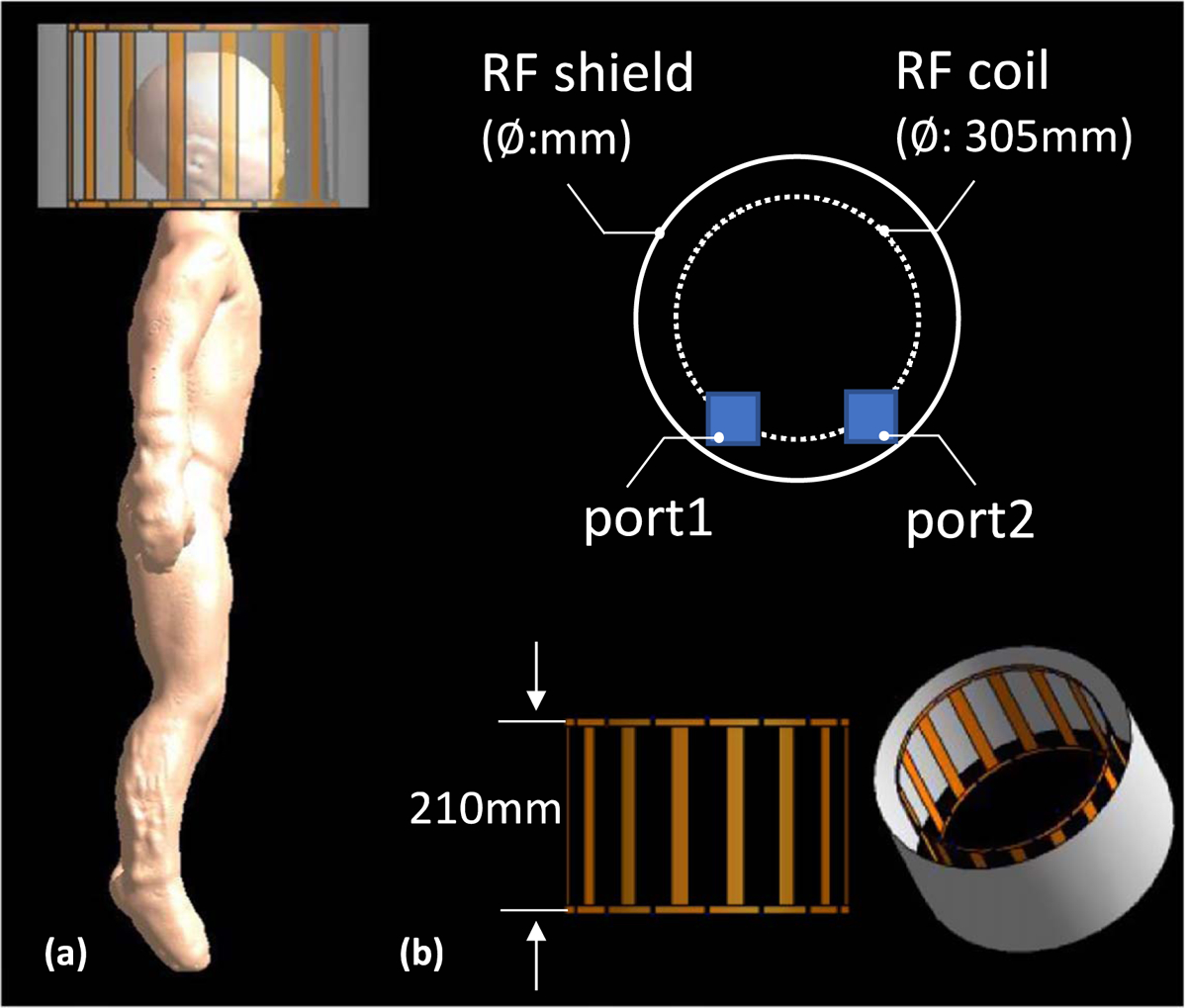
Electromagnetic simulation set-up. (a) A side view of the Athena model with its head centered inside a 7 T transmit coil. (b) Dimensions and the feeding port position of the 7 T head transmit coil—a 16-leg high-pass birdcage head transmit coil (coil outer diameter of 305 mm, coil length 210 mm, RF shield diameter: 372 mm).

Finite-difference time-domain solver (Yee 1966) was used to solve Maxwell’s equation at 298 MHz using Sim4Life. A 16-leg high-pass birdcage head transmit coil (Coil outer diameter of 305 mm, coil length 210 mm, RF shield diameter: 372 mm) was used as a commercially available 7 T head transmit coil (Clément *et al* 2022) (figure 6). The Athena model’s head was centered inside the head transmit coil to assess the electromagnetic (EM) field interaction with 3.5 year-old child tissue. An additional simulation with the Martin model (Jeong *et al* 2021a) was done in the same conditions for comparison. EM simulation results were normalized to 2 *μ*T at the coil center, which is the field strength to produce a 90 flip angle with 3 ms rectangular RF pulse (Collins and Smith 2001). For the thermal simulation, a structured time-domain thermodynamic solver in Sim4Life was used to solve Pennes’ bioheat equation. SAR is the power per unit mass deposited in the tissue and is defined by equation (1)

(1) $$\text{SAR}=\frac{\sigma \|E{\|}^{2}}{2\rho }\left({\text{W kg}}^{-1}\right),$$

where *σ* is the electrical conductivity (S m^−1^), *E* is the electric field (V m^−1^), and *ρ* is the mass density (kg m^−3^). The temperature (*T* in °C) of each tissue over time (*t* in s) was estimated using Pennes’ bio-heat partial differential equation (Pennes 1946) :

(2) $$\rho \cdot c\frac{\partial T}{\partial t}=\nabla \cdot (k\cdot \nabla T)+\rho \cdot Q-\rho_{b}c_{b}W(T)\cdot \left(T-T_{b}\right)+\rho \cdot SAR,$$

where *ρ* is the tissue mass density matrix (k m^−3^), *ρ*_*b*_ is the blood mass density (k m^−3^), ***c*** is the heat capacity matrix (J kg^−1^ °C^−1^), ***c***_*b*_ is the blood heat capacity (J kg^−1^ °C^−1^), *T* is the temperature matrix (°C), *T*_*b*_ is the basal blood temperature (°C), ***k*** is the thermal conductivity matrix (W m^−1^ °C^−1^), ***Q*** is the metabolic heat generation rate matrix (W kg^−1^), ***W*** (***T***) is the thermoregulated blood perfusion rate matrix (ml min^−1^ kg^−1^), and ***SAR*** is the specific absorption rate spatial peak matrix (W kg^−1^).

Virtual models’ equilibrium temperature in the MRI room (environment temperature: 23°C) was estimated by running the steady-state thermal simulation, and transient thermal simulation was conducted using the results of EM simulation as an input source for a 15 min MRI scan. The external air heat transfer rate was set to 6 W/(m^2^°C), and the internal air heat transfer rate was set to 10 W (m^2^°C). The reduced systemic thermoregulation (i.e. impaired model) was used as a conservative perfusion estimation (Jeong *et al* 2021b).

## 3. Results

**Figure 7.**
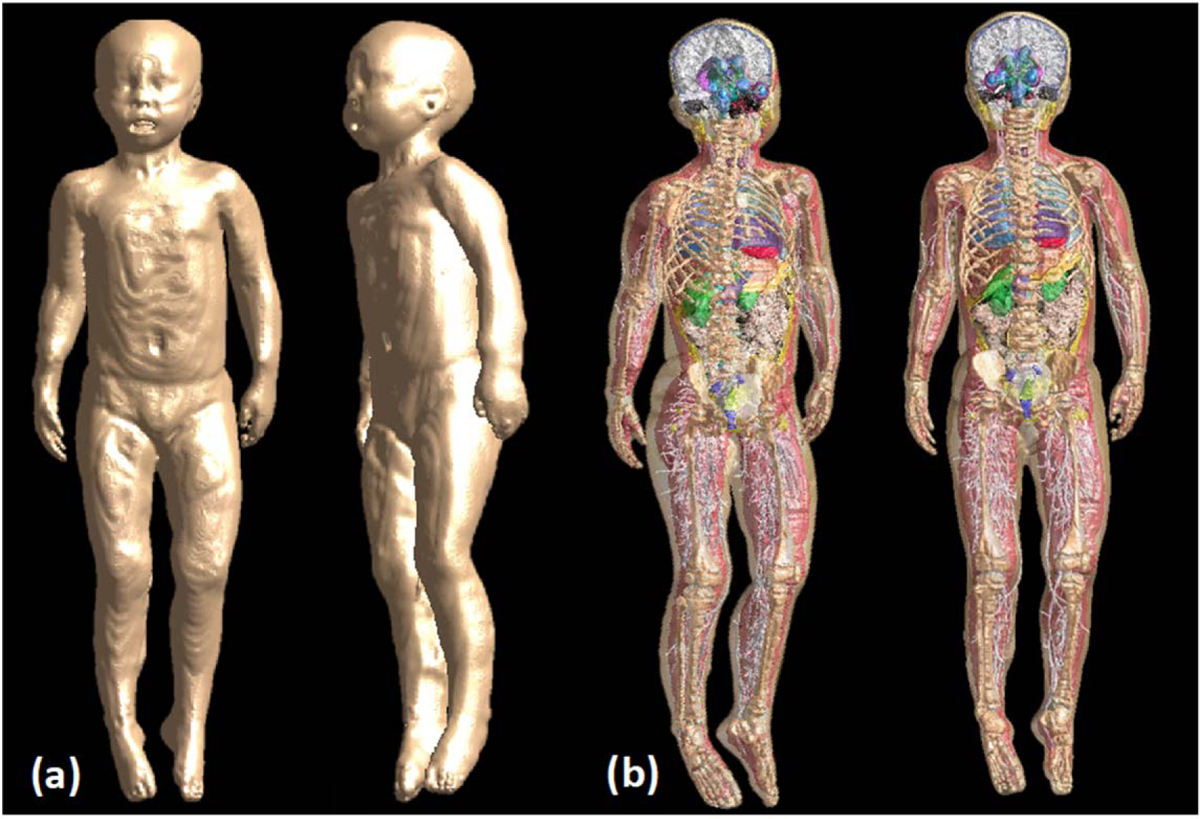
Athena, a 3.5 year-old female numerical model. (a) whole body surface on the coronal and left lateral view. The skin label represents the body surface. (b) The whole body with all tissues segmented, including skin, brain tissues, vessels, muscles, bones, and viscera visible on the right lateral and coronal views.

We used MRI sequences that covered the body from head to toe, including upper and lower extremities (i.e. T1 and IR), and sequences that focused on specific body parts (i.e. flair, magnetization-prepared rapid gradient-echo (MPRAGE) for the brain). Furthermore, CT and MRI were used for the torso. Athena, a representative 3.5 year-old female numerical model (figure 7), resulted in 267 tissue labels, each corresponding to a different anatomical tissue of the body (table 2, table S1).

### 3.1. Brain segmentation

**Figure 8.**
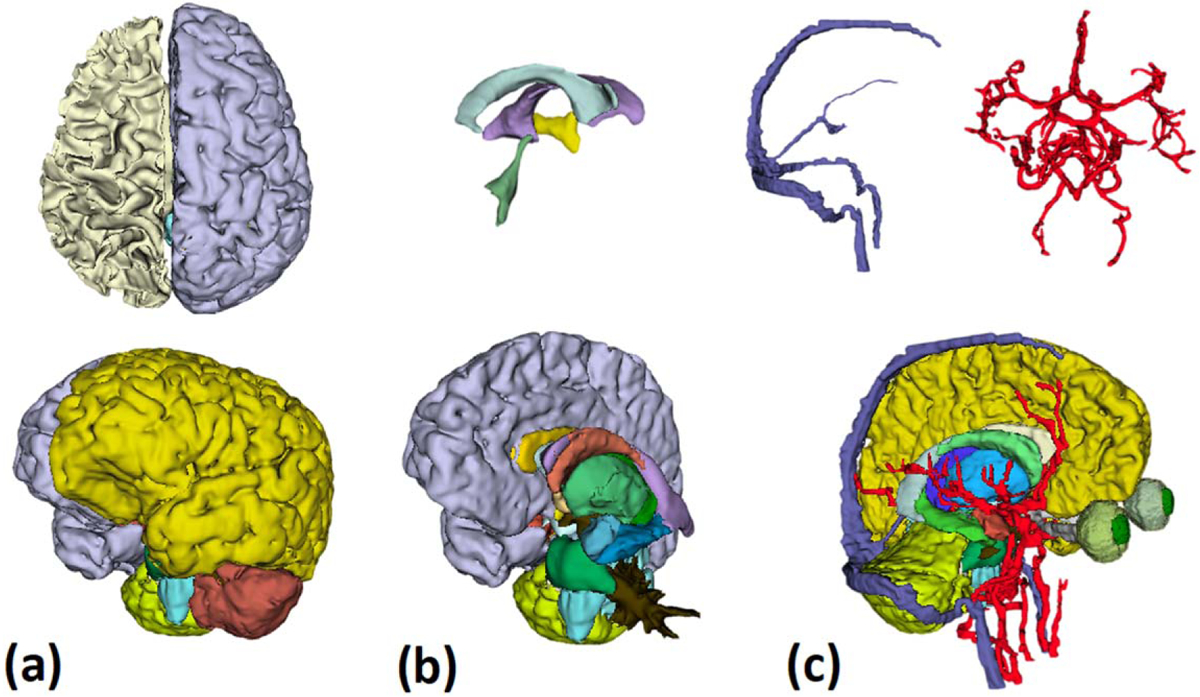
Brain structures segmentation: (a) brain structure segmentation using Infant Freesurfer, showing the cortical surfaces of the right and left brain-hemisphere and cerebellar lobes, as well as the white matter label of the left hemisphere. (b) The top half of the figure shows the brain’s ventricular system, while the lower part shows the deep brain structures as they were segmented using Infant Freesurfer. (c) Top: the brain’s vascular system (veins: left, arteries: right). Bottom: segmentation of the brain structures and the brain vessels after manual refinement and adding segmented tissues (e.g. optic nerves and optic chiasm).

Using automatic brain segmentation, we have segmented 26 different brain bilateral (i.e. left and right sides) tissues: cerebral and cerebellar grey and white matter, lateral ventricles, thalamus, caudate nucleus, putamen, globus pallidus, hippocampi, amygdala, nucleus accumbens, and ventral diencephalon. Furthermore, we also segmented six midline brain tissues: vermis, pons, medulla, midbrain, third and fourth ventricles. We manually refined these 32 automatically segmented tissues in order to achieve a higher level of anatomical accuracy. This refinement included but was not limited to the segmentation of white matter tracts of the cerebellum and vermis, sulci, and gyri, and the anterior and posterior genu of the internal capsule. As a result of the manual segmentation, we added six bilateral brain tissues: the optic nerves, cranial nerves (CN) (except CN2), the mammillary bodies, the choroid plexuses, the hypothalamus, and lateral ventricle CSF. We also added six midline tissues: the meninges, the optic chiasm, the vermis (white matter), third and fourth ventricles CSF and brain CSF (outside ventricles). In addition, we segmented the veins and the arteries that provide blood supply and drain the brain tissues, including the arterial circulation of the circle of Willis and its branches and the venous drainage, including the superior and inferior sagittal sinuses, the straight sinus, the transverse and sigmoid sinuses leading down to the jugular veins (figure 8). In addition, careful segmentation was performed on structures such as the external capsule, given that it represents white matter tracts separating two grey matter structures (putamen and insular cortex), leading to a total of 50 brain tissues.

### 3.2. Viscera and bone segmentation:

**Figure 9.**
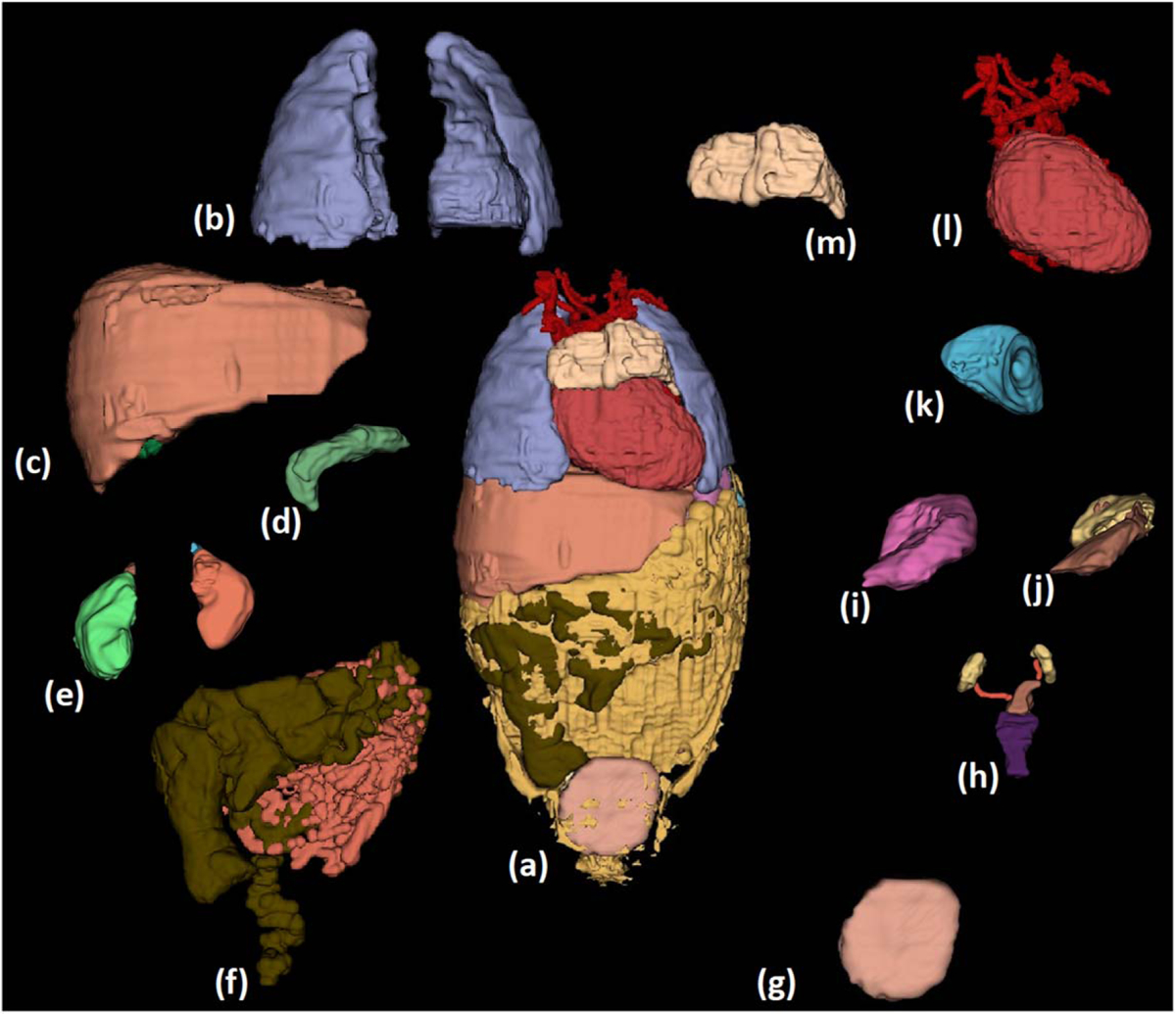
Chest and abdomen organ segmentation: (a) chest and abdomen all organs, (b) lungs, (c) liver (brown) and gallbladder (green), (d) pancreas (e) left (light brown) and right (green) kidneys and left (blue) and right (dark brown) adrenal glands (f) large bowel wall (dark brown), small bowel wall (orange) and intrabdominal fat (yellow), (g) urinary bladder (h) female genitalia (ovaries (yellow)), fallopian tubes (orange), uterus (brown), vagina (purple), (i) stomach wall, (j) stomach contents (brown), stomach air (yellow), (k) spleen and (l) heart muscle (brown) and vessels (red).

**Figure 10.**
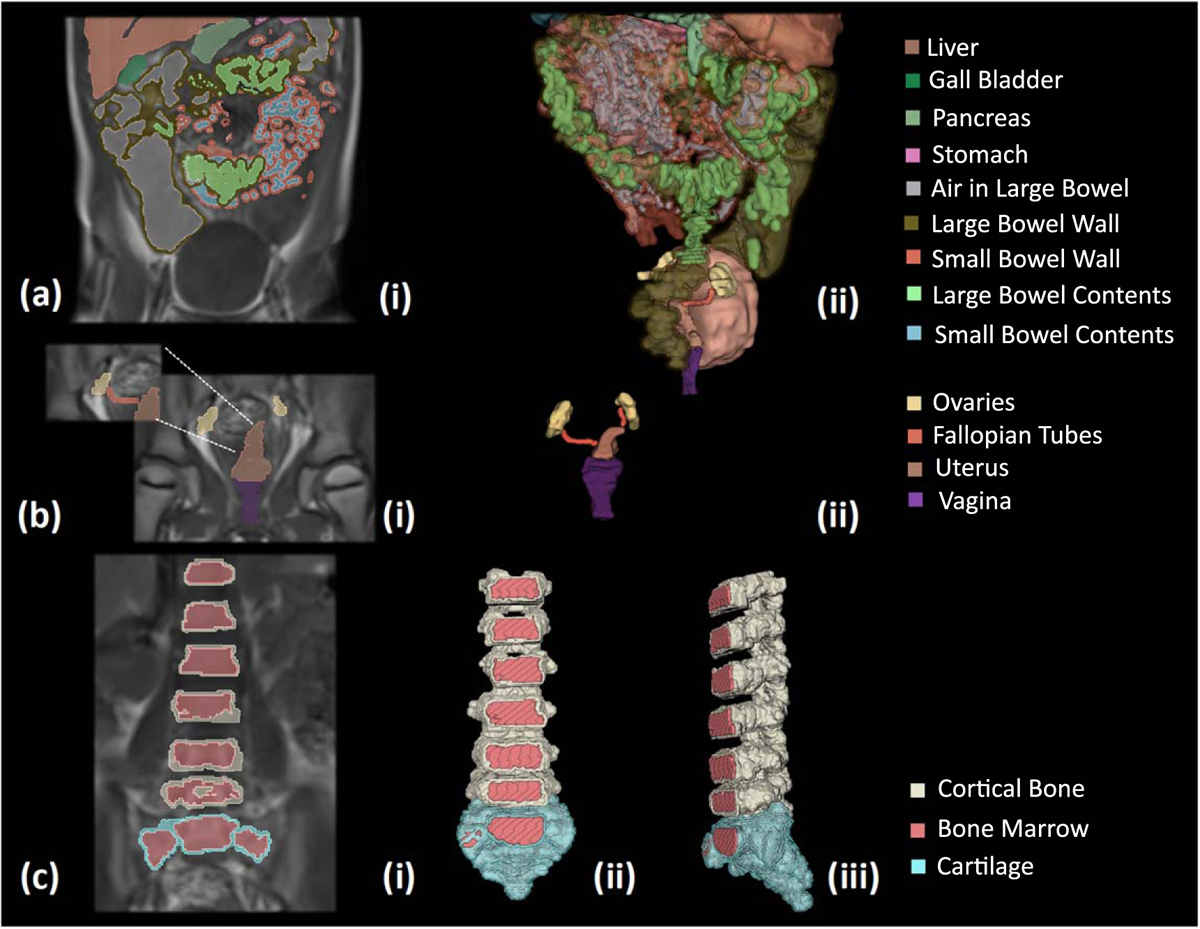
Segmentation and 3D representation of segmented tissues: (a) (i) segmentation of abdominal organs (viscera) on T1 MRI images in coronal plane and (ii) 3D opistholateral view of segmented tissues as reconstructed for the generation of the 3D model. (b) (i) Two different slices that show the segmentation of the female genitalia, including the ovaries, the fallopian tubes, the uterus, and the vagina on coronal MRI images, and (ii) 3D reconstruction of the segmented tissue. (c) Coronal MRI slice with the segmentation of the bone marrow, the cortical bone, and the cartilage of the sacral bone, the lumbar and lower two thoracic vertebrae. Colormap: each color corresponds to different segmented tissue. Not all the segmented tissues were included in this figure, but specific tissues were selected for visualization purposes.

**Figure 11.**
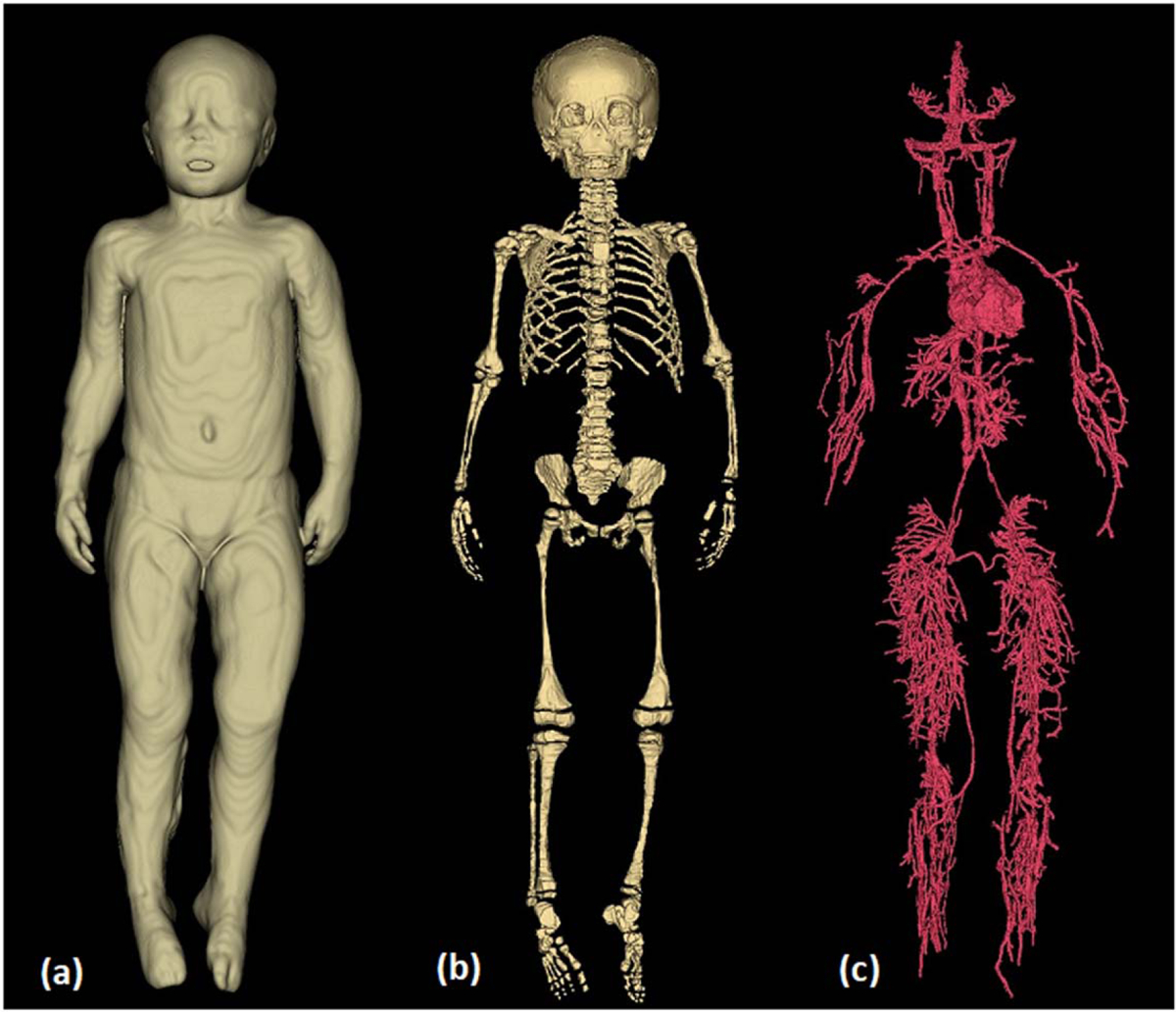
Whole body tissue segmentation for the development of a 3.5 year-old pediatric numerical model. (a) Whole body volume was segmented using a whole-body T1 MRI scan. (b) Whole-body bone segmentation. (c) Whole-body vessel segmentation, including extremities, head, and cardiac cavities based on T1 and IR images. MPRAGE and T2 Flair images were also used for the brain vessel segmentation.

The body’s organs were segmented using tissue-specific semi-automated techniques in the 3D Slicer. Following the process described in the flow diagram of figure 2, we segmented major organs of the head (e.g. eyes, nasal cavity, tongue, salivary glands), the neck (e.g. thyroid tissue, vessels, and trachea), the chest (e.g. thymus, heart, lungs, vessels, bones, cartilage), the abdomen (e.g. liver, gallbladder, pancreas, spleen, kidneys, adrenal glands, stomach, large and small intestines, air, bowel contents) and the pelvis (e.g. urinary bladder, vagina, uterus, fallopian tubes, ovaries) (figures 9 and 10(a), (b)). We used MRI images of T1, IR, and T2 Haste sequences to segment those tissues. For the structures of the chest, upper abdomen, and lower neck, we also used information from CT images to supplement the segmentation based on MR images. CT images of the chest were particularly useful in the segmentation of the great vessels of the heart. For the segmentation of the body’s vessels, T1 and particularly the IR sequences of the whole body, including the limbs, were highly significant. The vessels of the head and brain were differentiated into arteries and veins but not in the rest of the body (figures 8, 9 and 11). The bones of the skull estimated from an automated skull segmentation tool were manually refined, including the upper skull, the facial bones of the upper, and the lower mandible. Normal anatomy was closely followed, accounting for anatomical landmarks and cavities such as the nasal cavity. The rest of the body’s bones were segmented using a semi-automated supervised pipeline in 3D Slicer. This segmentation was based on identifying and segmenting the bone marrow, followed by the segmentation of the surrounding cortical bone, the cartilage in non-ossified parts of the body (i.e. sacral bone), and the joints (figures 10(c) and 11). All segmented tissues were referenced to the same 3D space of the full-body T1 MRI scan in order to avoid misalignments and overlap.

### 3.3. Validation

The internal validity of the segmentation process was tested using DSC, calculated for each of the three segmentors independently, and compared with the ground truth formulated by the two subspecialized neuroradiologists (table 3). An acceptable DSC above 0.8 was found for all 3 segmentors for all the 17 tissues (min: 0.81, max: 0.99, mean: 0.96, SD: 0.04). The primary segmentor (segmentor 3) had the highest score on DSC indicating higher concordance with the gold standard (segmentor 1, min:0.81 max: 0.99 mean: 0.95 SD: 0.05, segmentor 2, min:0.82 max: 0.99 mean: 0.95 SD: 0.05, segmentor 3, min: 0.9, max: 0.99, mean: 0.98 SD: 0.02). The highest match for all comparisons was found on the right lung and the liver (DSC: 0.99 for all three segmentors), followed by the right humerus and the tibia (1 versus GS: 0.98 2 versus GS: 0.99, 3 versus GS: 0.99) and the lowest at the gallbladder (1 versus GS: 0.86, 2 versus GS: 0.82, 3 versus GS: 0.94). (H-d, mm) showed similar results overall (min: 0.01, max: 2.09, mean: 0.26, SD: 0.4). Among individual tissues, the heart had the lowest H-d of 0.01 for segmentors 2 and 3 and 0.10 for segmentor 1, followed by the right humerus (1 versus GS: 0.11, 2 versus GS: 0.05, 3 versus GS: 0.01), while the lowest H-d was found on the segmentation of the bladder (1 versus GS: 1.66, 2 versus GS: 1.01, 3 versus GS: 0.57). In accordance with the DSC, segmentor 3 had the best performance (segmentor 1, min:0.04 max: 2.09 mean: 0.41 SD: 0.59, segmentor 2, min:0.01 max: 1.01 mean: 0.26 SD: 0.27, segmentor 3, min:0.01 max: 0.57 mean: 0.11 SD: 0.14).

The external validity (i.e. supported by literature) of the segmentation process was evaluated by comparing the segmented tissue properties, such as the weight, volume, or length depending on the tissue, with literature values (table 4). All the analyzed segmented tissues’ properties were within the literature values, except for the sternal bone and the CSF, which were smaller than the value. The spleen and the ovaries were also above the values suggested by ICRP Publ. 89 (ICRP 2002). Both lung weights were within the literature values, with the right lung weight of 238.5 g being heavier than the left lung weight of 159.7 g as expected according to the normal anatomy, given that the left thoracic space also accommodates the heart (ICRP 2002, Chang *et al* 2021). The brain weight was 1,084.8 g and had no deviation from the reference values (ICRP 2002, Chang *et al* 2021). The CSF had a volume of 104.5 ml, which was 5% smaller than reported in the literature (Matsuzawa *et al* 2001), perhaps since the meninges volume was segmented separately in Athena. The heart’s segmentation included only the heart muscle since the blood and the major blood vessels were segmented separately and weighed 82.4 g, which was within the literature values (ICRP 2002, Chang *et al* 2021). In addition, the right kidney weighed 62.7 g, and the left weighed 59.3 g, both within the literature values (OznurL *et al* 1998). The liver was 486.2 g, and its longest longitudinal diameter, measuring the right lobe, was 93.3 cm and within the literature values (OznurL *et al* 1998, ICRP 2002, Chang *et al* 2021). The large vessels running through the liver, such as the portal vein, were segmented separately.

Similarly, longest diameter was 72.6 cm, was within literature values while it is weight was in accordance with the values reported by Chang *et al* and 16% larger than the suggested values by ICRP Publ. 89 (OznurL *et al* 1998, ICRP 2002, Chang *et al* 2021). The stomach without contents weighed 42.9 g and the thymus weighed 30 g. These segmented tissues had no deviation from the values reported in the literature (Chang *et al* 2021). The ovaries without the fallopian tubes weighed 2.5 cm^3^ which is within the reported value by Chang *et al* and 16.7% larger than the suggested values by ICRP Publ. 89 (ICRP 2002, Chang *et al* 2021). The sternal bone was 6.1 cm, 1.4 cm shorter than the reference value (19.7%, see Discussion) (Weaver *et al* 2014), while the bone length ratios for the radius to the humerus, the tibia to the femur, the humerus to the femur, and the radius to the tibia were 0.76, 0.8, 0.7, and 0.68 respectively, all within the range reported in the literature (Robinow and Chumlea 1982) (table 4).

### 3.4. Brain segmentation metrics

Characteristics of the brain segmentation metrics of Athena and Martin, an age matched male model of the same age are presented in table S2. Cumulatively, the mean surface of Athena’s segmented brain regions is (4345.2, ±3340.1 mm^2^), gray matter volume is (15070.1, ±11626.2 mm^3^), gray matter thickness is (5.8, ±0.9 mm), curvature is (0.26, ±0.03 mm^−1^) and folding index is (84.9, ±68.1). Respectively for Martin, the mean surface of the same segmented brain regions is (3228.6, ±2323.4 mm^2^), gray matter volume is (13574.3, ±9972.9 mm^3^), gray matter thickness is (7.1, ±0.9 mm), curvature is (0.22, ±0.02 mm^−1^) and folding index is (65.1, ±62.9).

### 3.5. Tissue properties conversion and MRI RF safety simulation example

Table 5 shows the electrical (7 Tesla) and thermal tissue properties adjusted by age using an age-dependent conversion ratio. The cortical bone had the highest age-dependent conversion ratio in relative permittivity (i.e. 1.84) and electrical conductivity (e.g. 2.42), while conversion ratios for the skin and brain were 1.29 and 1.33 for the permittivity, and 1.47, and 1.51 for the electrical conductivity. At the same time, the brain showed the highest age-dependent conversion ratio for the basal tissue perfusion rate (e.g. 2.21), 1.49 for the skin, and 1.13 for the muscle (table 5). Similar tissue parameters were assigned for tissues with no measurement value (table S3).

**Figure 12.**
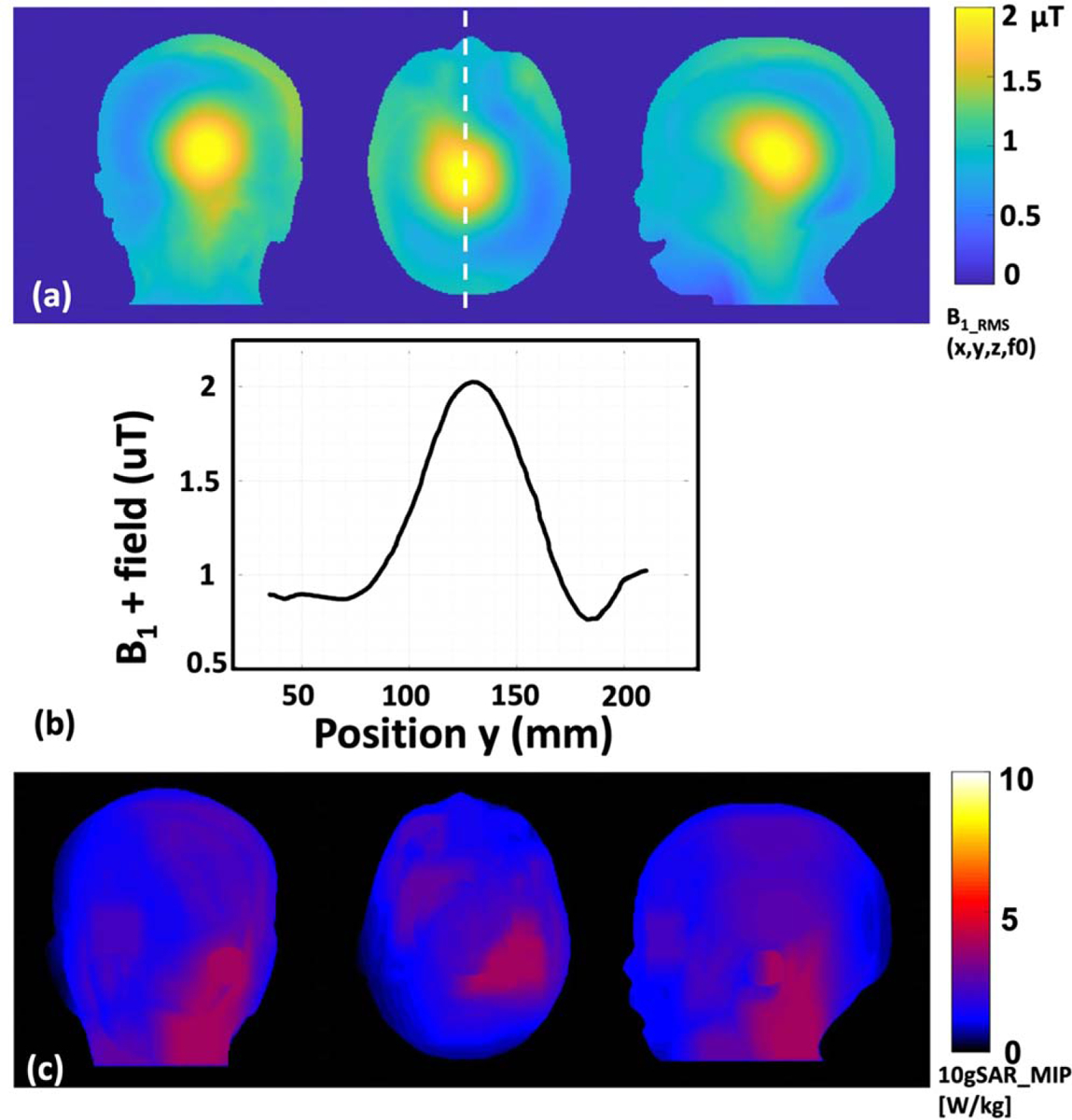
Electromagnetic simulation results: (a) coronal, axial, and sagittal view of the ${\text{B}}_{1}^{+}$ transmit magnetic field distribution in the head; (b) ${\text{B}}_{1}^{+}$ transmit magnetic field profile in the central line (dotted line of section (a)) of Athena’s head; (c) coronal, axial and sagittal view of the maximum intensity projection (MIP) of the 10 g mass averaged SAR in the head.

The results of EM simulation in ${\text{B}}_{1}^{+}$ transmit magnetic field distribution, and maximum intensity projection (MIP) of the 10 g-mass averaged SAR (10 g SAR) in Athena’s head are presented in figure 12. The RMS (root mean square) ${\text{B}}_{1}^{+}$ transmit magnetic field inhomogeneity in a 7 T MRI was estimated as expected when the head transmit coil was in a circularly polarized mode (figure 12(a)). The highest RMS ${\text{B}}_{1}^{+}$ transmit magnetic field was estimated as 2.03 *μ*T near the brain’s center, dropping rapidly to as low as 0.77 *μ*T in the peripheral region of the head (figure 12(b)). Table 6 shows the estimated SAR in a 7 T MRI with Athena and Martin. The maximum 10 g SAR was found was 3.95 W kg^−1^ in the Athena model (figure 12(c)), whereas the maximum 10gSAR estimated in the Martin model was 23% higher (i.e. 4.84 W kg^−1^, see table 6). In the thermal simulation, the maximum estimated temperature in Athena was 37.3 °C (figure S1), whereas a maximum temperature of 37.5 °C was estimated in Martin (0.5% difference) at the end of the 15 min scan.

## 4. Discussion

### 4.1. Data acquisition, preprocessing, and segmentation

Ideally, a numerical model should be developed using a multimodal approach (Iacono *et al* 2015) with techniques that precisely target each tissue compartment, for example, whole-body CT and MRI scans with multiple sequences with and without contrast. However, CT exposure in children is discouraged due to the presence of ionizing radiation (Pearce *et al* 2012, Mathews *et al* 2013), while lengthy MRI scans would require sedation for motion-artifact reduction (Edwards and Arthurs 2011), which would increase the risk of adverse events linked to anesthesia in children (Cravero *et al* 2009). The Boston Children’s Hospital (BCH) extensive database allowed us to identify a subject with whole-body MRI scans of different sequences and CT scans of specific body parts. Furthermore, the subject had the appropriate body characteristics (50th percentile for height and weight) to serve as a representative model for the 3.5 year-old healthy female population. The images used had no pathology and represented a healthy child of this age. Since the original MRI and CT scans were for clinical purposes, they were not acquired with iso-resolution. Thus, we resampled all images at 0.5 mm isotropic resolution, a technique also used for Martin’s development, and that allowed for minimizing the staircase phenomenon and maintaining high (submillimeter) resolution. All MRI images were acquired in the same session with minimal position changes, so the co-registration was straightforward.

On the other hand, even though CT images were acquired eleven months earlier than the MRI and that nonlinear registration was required, CT was used to guide the manual segmentation of the MRI images with poor contrast. For instance, the MR images of the heart were affected by motion artifacts, whereas the CT images were not to the same extent, revealing the location of the vessels’ insertion site in the heart. Furthermore, the CT delineated the intra-thoracic bone structures (i.e. sternum and clavicle) and bones of the ribcage (Jeong *et al* 2021a), which were not visible in the various MRI sequences.

For Athena’s segmentation, we used *in vivo* medical images with whole-body coverage MRI of one single subject (i.e. T1) with different sequences used for better imaging contrast of specific body areas such as the brain (e.g. T2 Flair) and the abdomen (e.g. T2 Half Fourier Single-shot Turbo spin-Echo (HASTE)), including CT images for the thorax not adopted in previously developed models. For instance, the UF Family (Lee *et al* 2005, 2010), and the XCAT (Norris *et al* 2014, Segars *et al* 2015) used scans with limited body coverage and filled the missing information with scans from different subjects or based their segmentation on CT scans, limiting anatomical detail and accuracy of the soft tissue such as the brain. In addition, our segmentation pipeline included no morphing of any of the body’s compartments. On the contrary, Nina from Virtual Population (Petoussi-Henss *et al* 2002, Gosselin *et al* 2014), the 5 year-old Chinese Family (Zhang *et al* 2009, Pi *et al* 2018), and the Korean child model (Lee *et al* 2009) all employed morphing that can significantly alter the anatomical accuracy of the model, given that childhood development and growth is a process with different paces for each body tissue. A typical example of this biological process is that at birth, the brain weighs ¼ of its adult size while the rest of the body is only 1/20 of its adult size, which indicates a different rate of development of the various non-parenchymal body parts (Burdi *et al* 1969). Furthermore, the multiple modalities and the automated and semi-automated segmentation tools resulted in a high resolution and detailed anatomical segmentation.

Athena was the product of a multi-step process of validation. The inter-segmentor comparisons with the DSC and the Hausdorff index (table 3), in addition to the validation by the expert neuroradiologists (figure 2) and the automated segmentation tools (figures 4 and 5), created a feedback loop that increased the anatomical accuracy of the final result. For all segmentors, a DSC index of 0.8 was achieved before finalizing each tissue compartment, while segmentor 3, the main segmentor, outperformed segmentors 1 and 2 for all tissues except the vagina and the uterus. Female genitalia at the early stages of life can be particularly challenging to visualize and outline, which might explain this discrepancy. The same results were found for the H-d, which was particularly helpful in evaluating the segmentation of large organs (e.g. liver, spleen) where the overlap between the different segmentors is expected to be higher, leading to a higher DSC value.

When measuring brain metrics, the differences that were found between the two models, might represent sex differences but can also reflect the age difference as well as individual characteristics of two different human subjects. Our results highlight that plurality and inclusion are highly valued in the human model generation, especially in an underrepresented and highly dynamic population such as young children.

Organ weight estimation and comparison with the age- and sex-specific values reported in the literature (Chang *et al* 2021) showed that the segmentation result was within the reference literature values for the lungs, the heart, the kidneys, the liver, the pancreas, the spleen, the stomach, the thymus and the ovaries and the brain. The literature values (Chang *et al* 2021) were estimated using ‘data to develop continuous relationships between physiological parameters and age, using a single form of mathematical equation. Four sets of equations (0–2 years male, 0–2 years female, 2–20 years male, 2–20 years female) for the body weight versus age, height versus age, and organ weight versus age relationships and 2 sets of equations (0–20 years male, 0–20 years female) for organ flow rate versus age relationship were developed. In regards to the remaining literature values, please refer to the detailed description in the references (Robinow and Chumlea 1982, OznurL *et al* 1998, Matsuzawa *et al* 2001, Kelsey *et al* 2013, Weaver *et al* 2014). The sternal bone is the only literature value without a range, and the percentual difference from the reference value (19.7%) is smaller than most other tissues ranges percentage of maximum compared to minimum. The left lung was approximately 30% smaller than the right lung, which is difference much greater than the 15% observed in adults ICRP. 89 (ICRP 2002). However, it is important to note that in children part of the left lung space is occupied by the thymus which is not present in adults. Therefore, if we add the weight of the thymus to the left lung, the difference between the left and the right lung is approximately 20%. The CSF was slightly below the reported values since the choroid plexuses occupied some space that would have otherwise been assigned to the CSF. In addition, detailed brain vessel segmentation, being more precise, with continuity requirements, and including more branches of the vascular system compared to Martin, had the same effect limiting the area otherwise occupied by the CSF. Finally, in their referenced work, Matsuzawa *et al* did not adjust the CSF volume occupied by the meninges, so a relative overestimation of the CSF space can be expected (Collins and Smith 2001). The spleen was found to be within and on the higher side of the values reported by Chang *et al* but 16% higher than the values suggested by the ICRP Publ. 89. This result might reflect eh high vascularity and blood content of this body tissue. A more detailed segmentation of the spleen’s vasculature would have resulted in lower net spleen mass. Although, given the tissue’s high vascularity and blood content this would have been of limited value in the current model while this could be considered in future versions. The ovaries followed a similar pattern with their volume being within the reported values by Kelsey *et al* (2013) and 16.7% larger than the values suggested by ICRP Publ. 89 (ICRP 2002). . This difference represents a 0.4 g difference in both ovaries combined, and could be attributed to the particularly challenging segmentation of these tissues given the complex anatomy of the pelvis and their relative size in prepubertal women of this young age. The sternal bone was 1.4 cm less than the reference value, which is explained by the variation in the ossification between the MRI and CT (performed first) scans that were 11 months apart, thus, the sternal bone, in reality, corresponds to a younger age. The bone length ratio, which also accounts for the internal validity of the bone segmentation process, was entirely in accordance with the literature values (table 4). In comparison with Martin, Athena was found to have higher weight/dimensions of all the measured organs (except the CSF and the sternal bones), given that Athena is 13 months older than Martin (Jeong *et al* 2021a). As previously noted, the sternal bones were segmented using CT when the subject was 11 months younger and thus smaller, and part of the CSF space was occupied by tissues that were not present in Martin’s model, therefore, CSF had a smaller volume. Finally, similar to the MARTIN model and the ICRP pediatric mesh-type reference computational phantoms (Choi *et al* 2021), the segmented voxel layers were converted to mesh-format which has benefits of data size reduction and improvements of anatomical representation for the complex organs such as lens of the eye (Gosselin *et al* 2014, Jeong *et al* 2021a).

### 4.2. The 7 T MRI simulation example

To the best of our knowledge, this is the first testing of a toddler/preschooler model in a 7 T MRI. Furthermore, no anatomically detailed open-source 3.5 year-old female model is available for computational simulation studies. Only one 7 T MRI pediatric safety study is present in the literature on a neonatal model (Clément *et al* 2022). In our comparison of sex as a biological variable, Martin had a 23% higher maximum 10gSAR compared to Athena. This difference was potentially due to sex and the head size of the two models. According to Le Garrec *et al* (2017). , a safety margin of 1.5 × is needed to account for inter-subject variability in MRI RF safety assessment which is within the difference in maximum 10gSAR between Martin and Athena models in 7 T head transmit coil. Similar to our findings, Clément *et al* (2022). also reported a 43.5% higher maximum 10gSAR in the Neonatal brain compared to the adult brain in 7 T with 8-channel transmit Dipole Array in Circular Polarized (CP) mode and a 2.43 fold higher maximum 10gSAR in the Neonatal brain compared to the adult brain in a 7 T head birdcage transmit coil in CP mode. Although direct comparison is not possible to assess the effect of the morphometric difference in a 7 T head MRI, Clement *et al*’s study used adult tissue properties for the simulation with the adult model and neonatal tissue properties for the neonatal model.

The simulation MRI RF results are within the safety limits prescribed by IEC 60601-3-33 (B:2015 I 60601 2 33 E 3 2010) standard. However, our example only illustrates how MRI safety studies can be conducted for children in a 7 T scanner. Further studies are needed as children under 66 pounds are not cleared by the FDA for 7 T MRI (U.S. Food and Drug Administration 2017).

### 4.3. Limitations

One limitation of the dataset used for the development of Athena is that no whole-body CT scan was available in the BCH database, and the CT images used were collected months before the MRI scan that identified the age of our subject. Having additional imaging studies with contrast would allow for a higher level of detailed segmentation of tissues such as the blood vessels, which had to be limited to the larger blood vessels of the body. In addition, our MRI/CT data were acquired at different resolutions in 3D space, which introduced Nyquist sampling issues. The out-of-plane MRI resolution ranged between 3.0 and 10 mm, while that of the brain provided either 1.1 mm or 3.0 mm (see table 1 for details on base image resolution). Thus, we adopted the Lanczos method in the data resampling process. However, future improvements in clinical MRI/CT of the out-of-plane resolution would lead to more accurate information.

The MRI images were acquired with a 3 T scanner that could provide a lower level of anatomical detail due to the lower signal-to-noise ratio (SNR) compared to more powerful scanners (e.g. 7 T). As a result, anatomical knowledge was applied to fill this gap in areas where the SNR was insufficient for segmenting smaller tissues, such as the meninges or small brain vessels. Establishing tissue boundaries was also a challenge for some organs. Although all scans were co-registered before the segmentation process and scan resolution was appropriate for most tissues, significant challenges in capturing and creating a realistic anatomical representation existed for moving tissues, such as the heart. In addition, variation in blood flow allowed only for limited segmentation of the body’s vessels, especially for the upper extremities. In addition, partial volume overlaps of specific body compartments such as CSF in some sequences such as the IR challenged the identification of the borders with other adjacent tissues. Despite the efforts for detailed segmentation of all identifiable body tissues, empty spaces were still found after completing the segmentation of the whole body. We used the ‘connective tissue’ label to fill those spaces as the most appropriate label given its position and role in the human body. Also, movement artifacts were found in some scans that included the extremities. Thus manual refinement using anatomical knowledge had to be applied. Furthermore, brain metrics of curvature, gray matter thickness, folding, and surface were calculated using the initial automatic segmentation result of FreeSurfer and did not account for the manual segmentation that was performed in both models. Although both models were manually refined, those changes are not expected to impact this comparison significantly.

## 5. Conclusions

This study presents a detailed, validated, and realistic 3.5 year-old female full-body open-source numerical model. A multi-step validation process was followed, including an expert’s opinion, comparing body metrics with literature reference values, and an already validated and published numerical model (i.e. Martin). Furthermore, Athena has a higher level of detail, giving more flexibility to the user of the model by including a separate label for each segmented bone and having different labels for structures that are symmetric in the human body, including the brain and the bones. Publicly available Athena can be used for many potential biomedical engineering studies, such as MRI RF dosimetry studies. In addition, we present an example of using the Athena model, which to the best of our knowledge, is the first toddler/preschooler MRI safety example in a 7 T head MRI and a comparison with Martin (Jeong *et al* 2021a), a male model, to study sex as a biological variable.

### 5.1. Sources of financial support

The research described in this paper was supported by the National Institutes of Health and the National Institute of Biomedical Imaging and Bioengineering NIH/NIBIB under the Grant R01EB024343.

## Supplementary Material

Supplementary material

## Figures and Tables

**Table 1.** MRI and CT sequence parameters used for segmentation.

Scans	Name of sequence	Voxel size (mm, mm, mm)	TR(ms)/TE(ms)/TI(ms)/FA(°)	FOV (mm)	NSA
MRI	AX IRI (head and neck)	0.94, 0.94, 4.00	2200/257/200/120	320	1
	AXIRII (chest)	1.09, 1.09, 4.00	3939.59/262/200/120	320	1
	AX IR III (abdomen and pelvis)	1.13, 1.13,4.00	2200.0/260/200/120	320	1
	AX/SAG/COR T1 MPRAGE (Brain)	1.03, 1.03, 1.10	1680.00/2.38/958/9	224	1
	AX T2 FLAIR (Brain)	0.43, 0.43, 3.00	9000/85/2500/150	416	1
	AX T2 HASTE (neck, chest and upper abdomen)	1.09, 1.09, 4.95	1600/97/0/142	320	1
	COR IR I (Head and neck)	1.48, 1.48,4.00	3860/53/220/120	256	1
	COR IR II (chest and abdomen)	1.48, 1.48, 5.00	3920/53/220/120	256	1
	COR IR III (pelvis and superior lower extremities)	1.56, 1.56, 5.00	3100/53/220/120	256	1
	COR IR IV (inferior lower extremities)	1.56, 1.56, 5.00	3100/53/220/120	256	1
	COR T1 (full body)	1.04, 1.04, 4.00	410/9.4/0/140	312	52
	COR T1 I(head, neck and upper chest)	1.06, 1.06, 4.00	410/9.4/0/75	320	1
	COR T1 II (neck, chest, abdomen and pelvis)	1.25, 1.25,5.00	400/11/0/90	320	1
	COR T1 III (pelvis and superior lower extremities)	1.88, 1.88,5.00	400/11/−1/90	320	1
	CORT1IV (inferior lower extremities)	1.04, 1.04, 5.00	997/9.5/−1/140	312	1
	SAG IR (lower spine)	0.63, 0.63, 3.00	2940/107/200/120	384	1
	SAG IR (upper spine)	0.63, 0.63, 3.00	2940/107/200/120	384	1
CT	AX Lung	0.37, 0.37, 10.00	0/0/0/0	512	1
	COR/SAG Lung	0.37, 0.37, 3.00	0/0/0/0	512	1
	TF Chest (2/5/6)	0.37, 0.37, 2.00/5.00/0.60	0/0/0/0	512	1

**Table 2.** Summary list of segmented tissues.

A. Bodytissues (torso)	Pancreas	C3 (C&BM)	Scapulae (L&R) (C&BM)	Cerebellar white matter (L&R)
Adrenal gland (L&R)	Retina (eyes) (L&R)	C4 (C&BM)	Skull	Cerebral greymatter (L&R)
Air head and neck	Sclera (L&R)	C5 (C&BM)	Sternum (C&BM)	Cerebral white matter (L&R)
Arteries head and neck	Skin	C6 (C&BM)	T1 (C&BM)	Choroid plexuses (L&R)
Air abdomen	Small bowel contents	C7 (C&BM)	T2 (C&BM)	Cranial nerves other (L&R)
Blood vessels body	Small bowel wall	Carpal bones (L&R) (C&BM)	T3 (C&BM)	CSF brain
Choroid (eye) (L&R)	Spinal cord	Cartilage	T4 (C&BM)	CSF spine
Ciliarymuscles (eye) (L&R)	Spleen	Clavicle (L&R) (C&BM)	T5 (C&BM)	Globus pallidus (L&R)
Connective tissue	Stomach wall	Femur (L&R) (C&BM)	T6 (C&BM)	Hippocampus (L&R)
Cornea (eye) (L&R)	Stomach contents	Fibula (L&R) (C&BM)	T7 (C&BM)	Hypothalamus (L&R)
Extraocular muscles (L&R)	Subcutaneous fat	Humerus (L&R) (C&BM)	T8 (C&BM)	Lateral ventricle (L&R) (CSF & meninges)
Fallopian tubes	Teeth erupted	Kneecap (L&R) (C&BM)	T9 (C&BM)	Mammillarybody(L&R)
Gallbladder	Teeth unerupted	L1 (C&BM)	T10 (C&BM)	Medulla
Heart muscle	Thymus	L2 (C&BM)	T11 (C&BM)	Meninges brain
Intrabdominal fat	Thyroid	L3 (C&BM)	T12 (C&BM)	Meninges spine
Kidney (L&R)	Tongue	L4 (C&BM)	Tibia (L&R) (C&BM)	Midbrain
Large bowel contents	Urinarybladder	L5 (C&BM)	Ulna (L&R) (C&BM)	Optic chiasm
Large bowel wall	Uterus	Lower mandible	Vertebral Discs	Optic nerve (L&R)
Lens (eye) (L&R)	Vagina	Metacarpal bones and Phalanges (L&R) (C&BM) (×5)	**C. Brain tissues**	Pons
Liver	Vitreous body (eye) (L&R)	Metatarsal bones and Phalanges (R&L) (C&BM)	3rd ventricle (CSF and meninges)	Putamen (L&R)
Lung (L&R)	Veins head and neck	Pelvic bone (C&BM)	4th ventricle (CSF and meninges)	Thalamus (L&R)
Lymphoid tissue (head)	**B. Bones**	Radius (L&R) (C&BM)	Accumbens (L&R)	Veins
Mucosa nasal cavity	Ankle bones (L&R) (C&BM)	Rib (L&R) (C&BM)	Amygdala (L&R)	Ventral diencephalon (L&R)
Muscles	C1 (C&BM)	Sacrum (C&BM)	Caudate (L&R)	Vermis greymatter
Ovaries	C2 (C&BM)	Rib (L&R) (C&BM)	Cerebellar greymatter (L&R)	Vermis white matter

* L&R: indicates that tissues were segmented as different labels for the right and left side *C&BM: cortex and bone marrow segmented separately as individual labels.

**Table 3.** Inter-operator availability between 3 segmentors across structures on the coronal MRI slices.

Tissue compartment	DSC			Hausdorff-distance (average, mm)		
	1 versus GT	2 versus GT	3 versus GT	1 versus GT	2 versus GT	3 versus GT
1. Gallbladder	0.86	0.82	0.94	0.55	0.70	0.28
2. Urinary bladder	0.88	0.92	0.96	1.66	1.01	0.57
3. Kidney left	0.96	0.96	0.98	0.34	0.39	0.18
4. Lung right	0.99	0.99	0.99	0.12	0.15	0.08
5. Liver	0.99	0.99	0.99	0.15	0.23	0.05
6. Heart muscle	0.96	0.99	0.99	0.10	0.01	0.01
7. Spleen	0.81	0.98	0.99	2.09	0.27	0.02
8. Uterus	0.99	0.86	0.95	0.04	0.35	0.14
9. Vagina	0.98	0.92	0.90	0.04	0.15	0.19
10. Humerus right	0.98	0.99	0.99	0.11	0.05	0.01
11. Tibia right	0.98	0.99	0.99	0.12	0.03	0.04
12. Femur right	0.94	0.92	0.99	0.35	0.49	0.01
13. Fibula right	0.96	0.99	0.99	0.80	0.01	0.01
14. Ulna right	0.97	0.95	0.99	0.08	0.16	0.02
15. Air nose, Sinuses	0.94	0.97	0.97	0.13	0.06	0.06
16. Vitreous body right	0.96	0.95	0.98	0.18	0.25	0.11
17. Radius right	0.97	0.97	0.99	0.16	0.16	0.01

**Table 4.** Validation table: weight measurements of representative segmented organ tissues and bone length rations according to age.

Tissue	Measurement type	Measured value	Literature value
Lung right	Weight (g)	238.5	120–320 (Chang *et al* 2021)^a^ 150–300 (ICRP 2002)^f^
Lung left	Weight (g)	159.7	120–320 (Chang *et al* 2021)^a^ (ICRP 2002)^f^
Brain	Weight (g)	1084.8	1000–1100 (Chang *et al* 2021)^a^ 950–1310 (ICRP 2002)^f^
Brain CSF	Volume (cm^3^)	104.5	110–120 (Matsuzawa *et al* 2001)^b^
Heart	Weight (g)	82.4	50–90 (Chang *et al* 2021)^a^ 50–85 (ICRP 2002)^f^
Kidneys (combined)	Weight (g)	79.46	70–110 (Chang *et al* 2021)^a^ (ICRP 2002)^f^
Kidney right	Length (cm)—longitudinal dimensions	62.7	40–70 (OznurL *et al* 1998)^c^
Kidney left	Length (cm)—longitudinal dimensions	59.3	40–70 (OznurL *et al* 1998)^c^
Liver	Weight (g)	486.2 330–570 (ICRP 2002)^f^	400–500 (Chang *et al* 2021)^a^
Length (cm)—longitudinal dimensions—right lobe	93.3	45–95 (OznurL *et al* 1998)^a^	
Pancreas	Weight (g)	23.2	up to 52 (Chang *et al* 2021)^a^ 20–35 (ICRP 2002)^f^
Spleen	Weight (g)	59.8 29–50 (ICRP 2002)^f^	30–60 (Chang *et al* 2021)^a^
Length (cm)—longitudinal dimensions	72.6	40–75 (OznurL *et al* 1998)^c^	
Stomach	Weight (g)	42.9	20–48 (Chang *et al* 2021)^a^ 20–50 (ICRP 2002)^f^
Thymus	Weight (g)	30	25–35 (Chang *et al* 2021)^a^ 30 (ICRP 2002)^f^
Ovaries	Volume (cm^3^)	2.5	0.6–3.6 (Kelsey *et al* 2013)
Weight (g)	2.4	0.8–2 (ICRP 2002)^f^	
Sternal bone	Length (cm) according to age	6.1	7.5 (Weaver*et al* 2014)^d^
Radius/humerus	Bone length ratio	0.76	0.71–0,78 (Robinow and Chumlea 1982)^e^
Tibia/femur	Bone length ratio	0.8	0.78–0.84 (Robinow and Chumlea 1982)^e^
Humerus/femur	Bone Length ratio	0.7	0.67–0.75 (Robinow and Chumlea 1982)^e^
Radius/tibia	Bone length ratio	0.68	0.61–0.70 (Robinow and Chumlea 1982)^e^

a 95% prediction interval.

b Range of volumes.

c Suggested normal longitudinal dimensions.

d Measurement of the superior-inferior dimension.

e Diaphyseal bone length ratio between the 5th–95th %ile.

f Mass in grams.

**Table 5.** Dielectric properties and tissue perfusion tissue of the 3.5 year-old female model at 7 Tesla.

Tissue properties at 297.2 MHz	Permittivity ratio^a^	3.5 year-old (y.o.) tissue permittivity	Conductivity ratio^b^	3.5 y.o. tissue conductivity(S m ^−1^)	Perfusion ratio^c^	Basal perfusion of 3.5 y.o. tissue (ml min^−1^ kg^−1^)
Adrenal gland	1.23	76.60	1.32	0.89	1.72	2505
Air	1.00	1.00	1.00	0.00	1.00	0
Bile	1.00	74.97	1.00	1.67	1.00	0
Blood	1.00	65.70	1.00	1.32	1.00	10 000
Blood vessel wall	1.24	60.04	1.36	0.73	1.00	150
Bone (Cortical)	1.84	24.78	2.42	0.20	1.72	17
Bone marrow(red)	1.24	15.07	1.36	0.24	1.72	232
Brain (grey matter)	1.33	79.79	1.51	1.04	2.21	1685
Brain (white matter)	1.33	58.19	1.51	0.62	2.21	468
Bronchi	1.24	56.28	1.36	0.83	1.72	409
Cartilage	1.24	58.11	1.36	0.75	1.72	409
Cerebellum	1.33	79.45	1.51	1.47	1.72	60
Cerebrospinal fluid	1.00	72.80	1.00	2.22	2.21	1699
Commissura anterior	1.33	58.19	1.51	0.62	0.00	0
Commissura posterior	1.33	58.19	1.51	0.62	2.21	468
Connective tissue	1.24	59.59	1.36	0.73	1.72	63.9
Diaphragm	1.20	69.62	1.36	1.05	1.72	170
Dura	1.33	63.72	1.51	1.21	2.21	838
Esophagus	1.24	85.34	1.36	1.32	1.72	326
Eye (aqueous humor)	1.00	72.80	1.00	2.22	1.00	0
Eye (cornea)	1.24	76.27	1.36	1.56	1.00	0
Eye (lens)	1.24	47.65	1.36	0.48	1.00	0
Eye (retina)	1.33	79.79	1.51	1.04	1.72	412
Eye (sclera)	1.24	73.16	1.36	1.33	2.21	838
Eye (vitreous humor)	1.00	69.02	1.00	1.52	0.00	0
Fat	1.24	14.58	1.36	0.10	1.72	56
Gallbladder	1.00	62.99	1.00	1.12	1.72	52
Heart muscle	1.20	82.99	1.36	1.23	1.13	1158
Hippocampus	1.33	79.79	1.51	1.04	2.21	1685
Hypophysis	1.23	76.89	1.32	1.13	2.21	1952
Hypothalamus	1.33	79.79	1.51	1.04	2.21	1685
Intervertebral Disc	1.24	58.65	1.36	1.24	1.72	60
Intestine contents	1.00	58.24	1.00	0.77	1.00	0
Kidney	1.24	87.67	1.36	1.39	1.21	4575
Large intestine	1.24	80.80	1.36	1.10	1.23	943
Larynx	1.24	58.11	1.36	0.75	1.72	60
Liver	1.21	64.93	1.25	0.76	1.20	1034
Lung	1.24	30.79	1.36	0.48	1.72	689
Mandible	1.84	24.78	2.42	0.20	1.72	17
Medulla oblongata	1.33	79.45	1.51	1.47	2.21	1232
Midbrain	1.33	79.45	1.51	1.47	2.21	1232
Mucous membrane	1.20	69.62	1.36	1.05	1.72	1020
Muscle	1.20	69.62	1.36	1.05	1.13	41
Nerve	1.24	45.88	1.36	0.57	1.72	275
Ovary	1.24	76.25	1.36	1.28	1.72	405
Pancreas	1.23	76.89	1.32	1.13	1.37	1049
Pineal body	1.23	76.89	1.32	1.13	2.21	1952
Placenta	1.00	65.70	1.00	1.32	1.72	2920
Pons	1.33	79.45	1.51	1.47	2.21	1232
Salivary gland	1.23	95.99	1.32	0.95	1.72	658
SAT (subcutaneous fat)	1.24	14.58	1.36	0.10	1.72	56
Skin	1.29	64.58	1.47	0.94	1.49	159
Skull cortical	1.84	24.78	2.42	0.20	1.72	17
Small intestine	1.24	86.76	1.36	2.50	1.23	1264
Spinal cord	1.24	45.88	1.36	0.57	1.72	275
Spleen	1.24	82.68	1.36	1.32	1.27	1972
Stomach	1.24	85.34	1.36	1.32	1.23	565
Tendon\ligament	1.24	59.59	1.36	0.73	1.72	50
Thalamus	1.33	79.79	1.51	1.04	2.21	1510
Thymus	1.24	66.67	1.36	0.00	1.72	424
Thyroid gland	1.23	76.89	1.32	1.13	1.72	9659
Tongue	1.24	73.16	1.36	1.01	1.13	88
Tooth	1.84	24.78	2.42	0.20	1.00	0
Trachea	1.24	56.28	1.36	0.83	1.72	60
Uterus	1.24	60.04	1.36	0.73	1.72	787
Urinary bladder Wall	1.24	24.96	1.36	0.43	1.72	134
Urine	1.00	49.95	1.00	1.75	1.00	0
Vagina	1.24	80.80	1.36	1.10	1.72	168
Vertebrae	1.84	24.78	2.42	0.20	1.72	17

a Permittivity ratio: permittivity of the 3.5 year-old tissue/permittivity of adult tissue.

b conductivity ratio: conductivity of the 3.5- year-old tissue/conductivity of adult tissue.

c Perfusion ratio: perfusion of a 3.5 year-old tissue/perfusion of adult tissue.

**Table 6.** Specific absorption rate and thermal simulation results of Athena and Martin in a 7 Tesla MRI with Tx/Rx head coil.

		Athena in a 7 T MRI with head Tx/Rx coil	Martin in a 7 T MRI with head Tx/Rx coil
EM simulation^a^	Head averaged SAR (W kg^−1^)	1.19	1.24
	Head maximum 10gSAR (W kg^−1^)	3.95	4.84
	Normalization factor (V)	44.45	44.63
Thermal simulation^b^	Maximum temperature in the head (°C)	37.29	37.45

a Fields were normalized to 2 *μ*T at the center of the coil.

b 15 min scan with fields normalized to 2 *μ*T at the coil center.
