# Supplementary material for: A high-resolution pediatric female whole-body numerical model with comparison to a male model
Source: Phys Med Biol. Author manuscript; Available in PMC 2023 Nov 3. (PMC10624254; doi:10.1088/1361-6560/aca950)
Supplement: Supplementary material [file NIHMS1935067-supplement-Supplementary_material.docx]

**Supplementary material:**

**Table S-1:** Analytic list of segmented tissues of Athena, a 3.5-year-old female numerical model.

| Accumbens (L) | Eyes Cornea (L) | L3 rib BM | Ovaries | T2 cortex |
| --- | --- | --- | --- | --- |
| Accumbens (R) | Eyes Cornea (R) | L4 cortex | Pancreas | T2 BM |
| Adrenal gland (L) | Eyes Lens (L) | L4 BM | Pelvic bone cortex | T3 cortex |
| Adrenal gland (R) | Eyes Lens (R) | L4 rib cortex | Pelvic bone BM | T3 BM |
| Air abdominal | Eyes muscles extraocular | L4 rib BM | Pons | T4 cortex |
| Air ears | Eyes Retina (L) | L5 cortex | Putamen (L) | T4 BM |
| Air neck | Eyes Retina (R) | L5 BM | Putamen (R) | T5 cortex |
| Air nose, sinuses | Eyes Sclera (L) | L5 rib cortex | R1 rib cortex | T5 BM |
| Amygdala (L) | Eyes Sclera (R) | L5 rib BM | R1 rib BM | T6 cortex |
| Amygdala (R) | Eyes Vitreous body (L) | L6 rib cortex | R2 rib cortex | T6 BM |
| Arteries, Circle of Willis | Eyes Vitreous body (R) | L6 rib BM | R2 rib BM | T7 cortex |
| C1 cortex | Fallopian tubes | L7 rib cortex | R3 rib cortex | T7 BM |
| C1 BM | Femur (L) Cortex | L7 rib BM | R3 rib BM | T8 cortex |
| C2 cortex | Femur (L) BM | L8 rib cortex | R4 rib cortex | T8 BM |
| C2 BM | Femur (R) cortex | L8 rib BM | R4 rib BM | T9 cortex |
| C3 cortex | Femur (R) BM | L9 rib cortex | R5 rib cortex | T9 BM |
| C3 BM | Fibula (L) cortex | L9 rib BM | R5 rib BM | T10 cortex |
| C4 cortex | Fibula (L) BM | L10 rib cortex | R6 rib cortex | T10 BM |
| C4 BM | Fibula (R) cortex | L10 rib BM | R6 rib BM | T11 cortex |
| C5 cortex | Fibula (R) BM | L11 rib cortex | R7 rib cortex | T11 BM |
| C5 BM | Foot Ankle (L) cortex | L11 rib BM | R7 rib BM | T12 cortex |
| C6 cortex | Foot Ankle (L) BM | L12 rib cortex | R8 rib cortex | T12 BM |
| C6 BM | Foot Ankle (R) cortex | L12 rib BM | R8 rib BM | Teeth (erupted) |
| C7 cortex | Foot Ankle (R) BM | Large bowel contents | R9 rib cortex | Teeth (unerupted) |
| C7 BM | Gallbladder | Large bowel wall | R9 rib BM | Thalamus (L) |
| Carpal bones hands (L) cortex | Globus Pallidus (L) | Lateral Ventricle (L) CSF | R10 rib cortex | Thalamus (R) |
| Carpal bones hands (L) BM | Globus Pallidus (R) | Lateral Ventricle (L) Meninges | R10 rib BM | Thymus |
| Carpal bones hands (R) cortex | Heart muscle | Lateral Ventricle (R) CSF | R11 rib cortex | Thyroid |
| Carpal bones hands (R) BM | Hippocampus (L) | Lateral Ventricle (R) Meninges | R11 rib BM | Tibia (L) cortex |
| Cartilage | Hippocampus (R) | Liver | R12 rib cortex | Tibia (L) BM |
| Caudate (L) | Humerus (L) cortex | Lower Mandible | R12 rib BM | Tibia (R) cortex |
| Caudate (R) | Humerus (L) BM | Lung (L) | Radius (L) cortex | Tibia (R) BM |
| Cerebral Gray Matter (L) | Humerus (R) cortex | Lung (R) | Radius (L) BM | Tongue |
| Cerebral Gray Matter (R) | Humerus (R) BM | Lymphoid tissue (head) | Radius (R) cortex | Ulna (L) cortex |
| Cerebral White Matter (L) | Hypothalamus (L) | Mammillary bodies (L) | Radius (R) BM | Ulna (L) BM |
| Cerebral White Matter (R) | Hypothalamus (R) | Mammillary bodies (R) | Sacrum cortex | Ulna (R) cortex |
| Cerebellum Gray Matter (L) | Intrabdominal fat | Medulla | Sacrum BM | Ulna (R) BM |
| Cerebellum Gray Matter (R) | Kidney (L) | Meninges brain | SAT (subcutaneous fat) | Urinary Bladder |
| Cerebellum White Matter (L) | Kidney (R) | Meninges spine | Scapulae (L) cortex | Uterus |
| Cerebellum White Matter (R) | Kneecap (L) cortex | Metacarpal bones & phalanges (L) cortex | Scapulae (L) BM | Vagina |
| Choroid plexus (L) | Kneecap (L) BM | Metacarpal bones & phalanges (L) BM | Scapulae (R) cortex | Veins brain/head |
| Choroid plexus (R) | Kneecap (R) cortex | Metacarpal bones & phalanges (R) cortex | Scapulae (R) BM | Ventral DC (L) |
| Clavicle (L) cortex | Kneecap (R) BM | Metacarpal bones & phalanges (R) BM | Skin | Ventral (DC (R) |
| Clavicle (L) BM | L1 cortex | Metatarsal bones (L) cortex | Skull | Vermis gray matter |
| Clavicle (R) cortex | L1 BM | Metatarsal bones (L) BM | Small bowel contents | Vermis white matter |
| Clavicle (R) BM | L1 rib cortex | Metatarsal bones (R) cortex | Small bowel wall | Vertebral discs |
| Connective tissue | L1 rib BM | Metatarsal bones (R) BM | Spinal cord | Vessels body |
| Cranial nerves other (L) | L2 cortex | Midbrain | Spleen | 3^rd^ ventricle CSF |
| Cranial nerves other (R) | L2 BM | Mucosa nasal cavity | Sternum cortex | 3^rd^ ventricle meninges |
| CSF brain | L2 rib cortex | Muscles | Sternum BM | 4^th^ ventricle CSF |
| CSF spine | L2 rib BM | Ophthalmic vein | Stomach contents | 4^th^ ventricle meninges |
| Eyes choroid (L) | L3 cortex | Optic chiasm | Stomach wall |  |
| Eyes choroid (R) | L3 BM | Optic nerve (L) | T1 cortex |  |
| Eyes Ciliary muscles | L3 rib cortex | Optic nerve (R) | T1 BM |  |

*L: Left; R: Right; BM: Bone Marrow; CSF: Cerebrospinal Fluid; DC: Diencephalon.

**Table S-2:** Brain metrics measurement of the 3.5-year-old female (Athena) and the 29-month-old male model (Martin).

|  | Athena | | | | | Martin | | | | |
| --- | --- | --- | --- | --- | --- | --- | --- | --- | --- | --- |
| Brain Structure | **Surface** | **GM Volume** | **GM Thickness** | **Curvature** | **Folding index** | **Surface** | **GM Volume** | **GM Thickness** | **Curvature** | **Folding index** |
| Bankssts | 1840 | 5131 | 5.568 | 0.192 | 16 | 1453 | 4600 | 6.5 | 0.202 | 17 |
| Caudal anterior cingulate | 919 | 3528 | 6.247 | 0.25 | 17 | 982 | 3187 | 6.043 | 0.227 | 11 |
| Caudal middle frontal | 3606 | 12702 | 5.703 | 0.266 | 71 | 3001 | 12446 | 6.904 | 0.217 | 48 |
| Cuneus | 2412 | 6422 | 4.605 | 0.313 | 55 | 1819 | 4072 | 6.532 | 0.244 | 48 |
| Entorhinal | 589 | 3289 | 7.366 | 0.252 | 9 | 533 | 2219 | 5.5 | 0.213 | 15 |
| Fusiform | 4829 | 17152 | 5.922 | 0.266 | 105 | 3762 | 17434 | 7.394 | 0.233 | 78 |
| Inferior parietal | 9874 | 31856 | 5.499 | 0.247 | 182 | 6365 | 28216 | 7.231 | 0.225 | 134 |
| Inferior temporal | 5618 | 24318 | 6.451 | 0.274 | 126 | 3813 | 17101 | 7.12 | 0.227 | 307 |
| Isthmus cingulate | 1774 | 6283 | 5.351 | 0.26 | 37 | 1512 | 5172 | 6.321 | 0.223 | 18 |
| Lateral occipital | 9351 | 26080 | 4.948 | 0.289 | 211 | 5771 | 20580 | 6.039 | 0.26 | 139 |
| Lateral orbitofrontal | 4140 | 15619 | 6.471 | 0.264 | 75 | 2516 | 12387 | 8.22 | 0.21 | 32 |
| lingual | 4800 | 12359 | 4.631 | 0.302 | 106 | 3578 | 15870 | 6.948 | 0.23 | 78 |
| Medial orbitofrontal | 2764 | 11014 | 6.378 | 0.24 | 57 | 1916 | 9711 | 8.066 | 0.208 | 32 |
| Middle temporal | 5819 | 25942 | 6.467 | 0.256 | 111 | 4220 | 23480 | 7.883 | 0.23 | 90 |
| Para-hippocampal | 1253 | 4039 | 5.707 | 0.238 | 14 | 1158 | 5801 | 7.706 | 0.208 | 16 |
| Para-central | 2626 | 9065 | 5.758 | 0.262 | 39 | 2222 | 7245 | 5.998 | 0.204 | 28 |
| Pars opercularis | 2244 | 7980 | 5.871 | 0.229 | 33 | 2054 | 9233 | 7.498 | 0.209 | 34 |
| Pars orbitalis | 1260 | 5914 | 6.421 | 0.301 | 36 | 855 | 5278 | 8.021 | 0.257 | 23 |
| Pars triangularis | 2304 | 8782 | 5.865 | 0.251 | 41 | 2293 | 11624 | 7.779 | 0.22 | 45 |
| Pericalcarine | 1962 | 2769 | 3.176 | 0.282 | 34 | 1898 | 4493 | 5.076 | 0.22 | 25 |
| Postcentral | 7600 | 21399 | 4.651 | 0.25 | 142 | 6089 | 19348 | 5.618 | 0.213 | 100 |
| Posterior cingulate | 1895 | 6497 | 5.869 | 0.274 | 41 | 1586 | 6373 | 6.903 | 0.234 | 25 |
| Precentral | 8645 | 25846 | 5.084 | 0.242 | 127 | 6564 | 23654 | 6.343 | 0.217 | 98 |
| Precuneus | 7236 | 23735 | 5.624 | 0.26 | 138 | 4632 | 18829 | 6.876 | 0.215 | 85 |
| Rostral anterior cingulate | 1114 | 4203 | 5.915 | 0.226 | 26 | 767 | 3270 | 7.207 | 0.173 | 7 |
| Rostral middle frontal | 9375 | 35618 | 5.788 | 0.29 | 221 | 6747 | 31006 | 7.235 | 0.235 | 152 |
| Superior frontal | 11331 | 46866 | 6.584 | 0.271 | 227 | 8923 | 40911 | 7.539 | 0.22 | 139 |
| Superior parietal | 11140 | 33844 | 4.969 | 0.256 | 222 | 6963 | 25314 | 6.253 | 0.226 | 136 |
| Superior temporal | 6950 | 26271 | 6.11 | 0.213 | 106 | 5443 | 23996 | 7.343 | 0.199 | 89 |
| Supramarginal | 6615 | 22868 | 5.565 | 0.266 | 144 | 5626 | 25970 | 7.497 | 0.223 | 113 |
| Frontal pole | 499 | 3057 | 6.977 | 0.345 | 21 | 335 | 3184 | 9.11 | 0.251 | 12 |
| Temporal pole | 818 | 4737 | 7.395 | 0.283 | 18 | 445 | 4375 | 8.997 | 0.213 | 14 |
| Transverse temporal | 771 | 2093 | 4.899 | 0.276 | 15 | 602 | 2493 | 7.049 | 0.196 | 7 |
| insula | 3765 | 15106 | 6.897 | 0.229 | 64 | 3330 | 12653 | 7.378 | 0.162 | 18 |

Brain structures are based on the Desikan-Kylianny brian anatomical atlas.

GM: Gray Matter, Bankssts: represents cortical areas around the superior temporal sulcus

**Table S-3**. Tissue property assignment for the tissues for which no measurements have been published. Tissue categorization rule is followed by Virtual Population[1] and MIDA head model [2]. Also, see **Table 5** for the converted tissue properties for 3.5-year-old tissue.

| Database | Tissues in Martin |
| --- | --- |
| Adrenal Gland | Adrenal Gland |
| Air | Air Head, Air Neck |
| Bile | Bile |
| Blood | Blood, Heart Lumen |
| Blood Vessel Wall | Penis, Ureter, Urethra |
| Bone (Cortical) | Bone (Cortical), Skull Cortical, Tooth, Rib and Vertebrae (Cortical) |
| Bone Marrow (Red) | Bone Marrow Red, Skull (Bone Marrow Red) Vertebrae and Rib (Bone Marrow Red) |
| Brain (Grey Matter) | Brain (Grey Matter), Pallidum, Accumbens Area, Substantia Nigra, Amygdala, Mammillary body, Caudate, Putamen, Eye (Retina), Hippocampus, Hypothalamus, Thalamus |
| Brain (White Matter) | Brain White Matter, Cerebellum White Matter, Vermis White Matter |
| Cartilage | Nasal Cartilage, Long bones joint and femur cartilage, Secondary ossification centers in long bones |
| Cerebellum | Cerebellum, Medulla Oblongata, Midbrain, Pons, Vermis Grey Matter |
| Cerebrospinal Fluid | Cerebrospinal Fluid, CSF Spinal Cord, Eye (Aqueous Humor) |
| Dura | Meninges Brain, Meninges Spinal Cord |
| Esophagus | Esophagus |
| Eye (Aqueous Humour) | Eye Aqueous Humour |
| Eye (Cornea) | Eye (Cornea) |
| Eye (Lens) | Eye (Lens) |
| Eye (Sclera) | Eye (Sclera) |
| Eye (Vitreous Humour) | Eye (Vitreous Humour) |
| Fat (Average Infiltrated) | Fat, Subcutaneous Fat (SAT) |
| Gallbladder | Gallbladder |
| Heart Muscle | Heart Muscle |
| Intervertebral Disc | Intervertebral Disc |
| Intestine contents (10) | Large Intestine contents, Small intestine contents |
| Kidney | Kidney |
| Large Intestine | Large Intestine |
| Liver | Liver |
| Lung | Lung |
| Muscle | Muscle, Muscle periocular, Eye muscle, Mucous Membrane |
| Nerve | Optic nerves, Cranial Nerves (Large Branches II V VIII IX), Spinal Cord |
| Pineal Body | Pineal gland |
| Salivary Gland | Salivary Gland |
| Skin | Skin |
| Small Intestine | Small Intestine |
| Spleen | Spleen |
| Stomach | Stomach, Esophagus |
| Tendon\Ligament | Connective Tissue, Tendon\Ligament |
| Testis | Epididymis, Prostate, Seminal vesicle, Testis |
| Thymus | Thymus |
| Thyroid gland | Hypophysis, Pancreas, Pineal Body, Thyroid Gland |
| Tongue | Tongue |
| Trachea | Bronchi, Trachea |
| Trachea Lumen | Trachea Lumen |
| Urinary bladder | Urine |
| Uterus | Uterus |

**Figure S-1: Thermal simulation results**. Coronal and sagittal view of the thermal simulation results of 15-minutes of MRI scan with field normalized to 2µT at the center of the 7T head transmit coil using Athena, a 3.5-year-old female numerical model.

**Formula S-1: Tissues properties conversion formula.**

$3years human tissue properties = Adult human tissue properties \times\frac{{Rat}_{10days}}{{Rat}_{adult(70 days)}}$

**References:**

1. Gosselin MC, Neufeld E, Moser H, et al. Development of a new generation of high-resolution anatomical models for medical device evaluation: The Virtual Population 3.0. *Phys Med Biol*. 2014;59(18):5287-5303. doi:10.1088/0031-9155/59/18/5287

2. Iacono MI, Neufeld E, Akinnagbe E, et al. MIDA: A multimodal imaging-based detailed anatomical model of the human head and neck. *PLoS One*. 2015;10(4). doi:10.1371/journal.pone.0124126
